# Construction of an integrative regulatory element and variation map of the murine *Tst* locus

**DOI:** 10.1186/s12863-016-0381-6

**Published:** 2016-06-11

**Authors:** Jasmina Beltram, Nicholas M. Morton, Tanja Kunej, Simon Horvat

**Affiliations:** Biotechnical Faculty, Animal Science Department, University of Ljubljana, Groblje 3, 1230 Domzale, Slovenia; Molecular Metabolism Group, University/British Heart Foundation Centre for Cardiovascular Science, University of Edinburgh, Queen’s Medical Research Institute, 47 Little France Crescent, Edinburgh, EH16 4TJ UK; National Institute of Chemistry, Hajdrihova 19, 1000 Ljubljana, Slovenia

**Keywords:** Thiosulfate sulfurtransferase (TST), Rhodanese, Regulatory elements, Map, Obesity, Lean, Gene variants, Polymorphism, Bioinformatics

## Abstract

**Background:**

Given the abundance of new genomic projects and gene annotations, researchers trying to pinpoint causal genetic variants are faced with a challenging task of how to efficiently integrate all current genomic information. The objective of the study was to develop an approach to integrate various genomic annotations for a recently positionally-cloned *Tst* gene (Thiosulfate Sulfur Transferase, synonym Rhodanese) responsible for the *Fob3b2* QTL effect on leanness and improved metabolic parameters. The second aim was to identify and prioritize *Tst* genetic variants that may be causal for the phenotypic effects.

**Results:**

A bioinformatics approach was developed to integrate existing knowledge of regulatory elements of the *Tst* gene. The entire *Tst* locus along with flanking segments was sequenced between our unique polygenic mouse Fat and Lean strains that were generated by divergent selection on adiposity for over 60 generations. The bioinformatics-generated regulatory element map of the *Tst* locus was then combined with genetic variants between the Fat and Lean mice and with comparative analyses of polymorphisms across 17 mouse strains in order to prioritise likely causal polymorphisms. Two candidate regulatory variants were identified, one overlapping an evolutionary constrained *Tst* intronic element and the other residing in the seed region of a predicted 3′UTR miRNA binding site.

**Conclusions:**

This study developed a map of regulatory elements for the *Tst* locus in mice and identified candidate genetic variants with increased causal likelihood. This map provides a basis for experimental validation and functional analyses of this novel candidate leanness and antidiabetic gene. Our methodological approach is of general utility for analyzing regulation of loci that have limited annotations and experimental evidence and for identifying candidate causal regulatory genetic variants in post-GWAS or post-QTL- cloning studies.

**Electronic supplementary material:**

The online version of this article (doi:10.1186/s12863-016-0381-6) contains supplementary material, which is available to authorized users.

## Background

In the last decade we have witnessed intense efforts to identify genetic variants controlling complex traits in various species. In humans, for example, genome wide association studies (GWAS) have identified several thousand gene variants associated with complex traits and diseases [1]. These gene variants can include structural variants such as insertion/deletions, in-frame deletions (indels), inversions, copy-number variants (CNVs) and most frequently (over 95 %) single nucleotide polymorphism (SNP) markers [2, 3]. A majority of variants mapped to candidate regions are unlikely to be causal for the effect on the trait [4]. They likely represent closely linked genetic markers that are in linkage disequilibrium (LD) with the causal variant. In animal models loci for complex traits (quantitative trait loci; QTL), can also be detected using GWAS approaches, especially in outbred species. However, in inbred laboratory animal models complex trait loci are more frequently identified using genetic analyses of crosses between strains differing in the trait(s) of interest. Animal models offer additional complementary methods for identifying candidate genes and their related pathways in humans. Advantages of using animal genetic models are the ability to better control environmental factors, genetic models are less heterogeneous, and gene expression can be examined in some tissues not readily available from humans. Finally, genes found for complex traits in animal models are often conserved in humans (e.g., [5–7]).

A major hurdle in GWAS or QTL studies is to proceed efficiently from detected markers linked to the candidate loci of interest to identification of the causal genetic variant responsible for the phenotypic effect. This holds true also for cases where the locus responsible for a QTL effect has been identified (e.g. by a combination of genetic mapping, expression and transgenic studies) but the genetic variant responsible within the locus has not been identified. Until recently, efficient prioritisation of candidate genetic variants based on bioinformatics analyses was hindered by limited functional annotation of the genome, especially outside coding sequences. However, in the recent years, several large-scale genomics projects such as Encyclopedia of DNA Elements (ENCODE; [8]), and the Functional Annotation of the Mammalian Genome (FANTOM; [9]), as well as improved genomic prediction tools now provide a comprehensive functional annotation in various cell and tissue types as well as developmental stages [8, 10–13]. Major advances in functional annotations are found in novel transcripts, promoters, enhancers, insulators, chromatin modification sites, transcription factor binding sites, as well as inter-species and intra-species conserved DNA elements.

Given the flood of new genomic projects and annotations, researchers looking for the best candidate causal genetic variants are faced with a challenging task of how to efficiently integrate all current genomic information. Although some genomic databases such as Ensembl [14] combine information from several different genome annotation projects, they still lack some information and tools to explore in detail a particular segment of the genome.

Our unique polygenic Lean (L) and Fat (F) mouse models have been described in detail in previous studies [15–17]. Briefly, original F and L lines were generated by divergent selection on adiposity for over 60 generations and at the end differed in body fat percentage by more than five-fold (Fat line, 23 % body fat, Lean line, 4 % body fat) [15, 16, 18]. Genetic studies have shown that the two polygenic mouse lines represent an excellent model for identifying genetic factors underlying the complex human obesity and leanness mechanisms. Several major quantitative trait loci (QTL) underlying fat divergence have already been described [11, 19, 20] and some QTLs have been further mapped to a higher resolution and eventually positionally cloned [21, 22]. In the present study we focus on the nuclear-encoded mitochondrial thiosulfate sulfur-transferase (*Tst*, also known by synonym Rhodanese) that we recently identified in a positional cloning experiment as a causal gene for the *Fob3b2* QTL phenotypic effect [22]. However, the genetic variants responsible for upregulation of *Tst* expression in the Lean mice have not yet been identified.

The main objective of the present study was to integrate various genomic annotations in the *Tst* locus to construct a map of regulatory elements of this gene and to identify and prioritise the causal genetic variants between the Fat and Lean lines. We focused on regulatory elements because the main driver of the phenotypic effect on leanness and metabolic parameters was an increase in expression of *Tst* in Lean compared to Fat mice [22]. To first uncover polymorphisms that may be causal for the difference in expression of the *Tst* gene, we undertook a classical high resolution Sanger-sequencing of the entire *Tst* locus in Fat and Lean lines. The identified genetic variants were then evaluated and prioritised using our regulatory element map of the *Tst* locus that integrated broad functional information from conserved polymorphisms in other strains, association studies, transcription factor binding site motifs, chromatin modification motifs and miRNA binding sites. Our approach to build a detailed integrative regulatory element map is of general utility as it can be applied for evaluating and prioritising polymorphisms within candidate regions in any trait or species of interest. Reducing and prioritising the number of potential causal polymorphisms is essential for efficient planning of further experiments to prove or support causality of candidate genetic variants. This may ultimately answer important basic research questions as well as provide a foundation for potential therapeutic developments.

## Methods

### Bioinformatics analysis –criteria for defining high priority regulatory sites

**Fig. 1:**
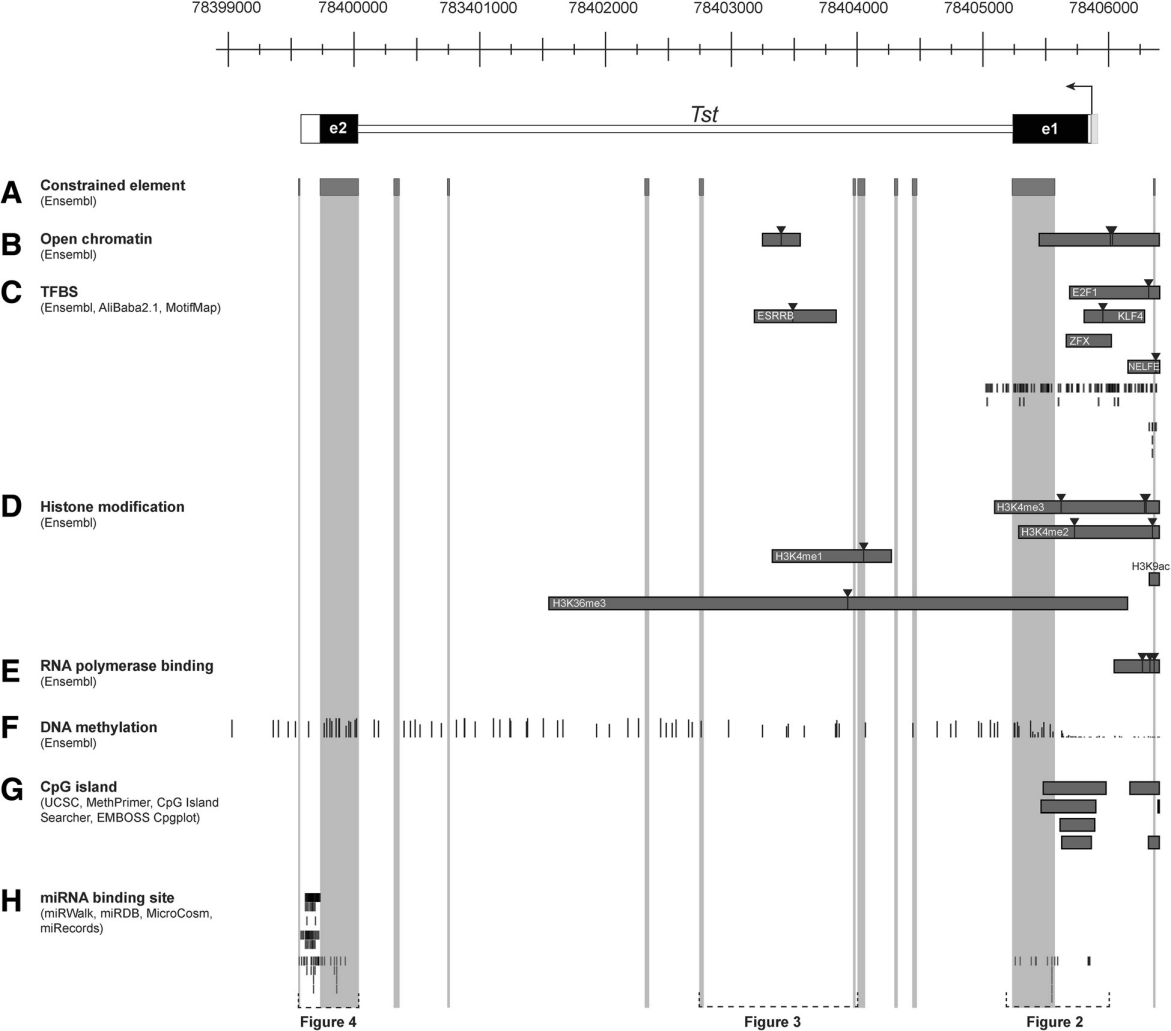
Transcription regulatory element atlas for *Tst* gene. Schematic presentation of predicted and experimentally validated data about mouse *Tst* transcription regulation, obtained from various genomics resources. *Tst* gene structure: white boxes – 3′ and 5′ UTR; black boxes – exons; white line – intron region. **a** Grey boxes show constrained elements betwen 39 Eutherian mammals. **b** DNAseI sites are presented as grey boxes and peaks (inverted triangles). **c** Black lines present predictions of TF binding sites. Grey stripes present ENCODE data with peaks (inverted triangles) for TFBS (**c**) histone modifications (**d**) and RNA polymerase binding sites (**e**). **f **The degree of DNA methylation is presented as a height line (100% methylation - the heighest line, 0% methylation - the lowest line). **g** Grey stripes present predicted CpG islands from various prediction tools. **h** Black lines display predicted miRNA target sites

Bioinformatics analysis was performed on a 7400 bp-long segment of the mouse *Tst* gene, including the ~ 0.5 kb upstream and downstream regions (Fig. 1; Additional file 1: Table S1, Additional file 2: Table S2, Additional file 3: Table S3, Additional file 4: Table S4, Additional file 5: Table S5, Additional file 6: Table S6, Additional file 7: Table S7, Additional file 8: Table S8, Additional file 9: Table S9, Additional file 10: Table S10). To define the high priority regulatory segments of the *Tst* gene we applied the following criteria – a) presence of at least one evolutionary constrained element b) a minimum of two other regulatory features such as open chromatin, transcription factor binding site, histone modification, RNA polymerase binding site, DNA methylation, CpG island or microRNA (miRNA) binding sites. These regulatory features were identified and mapped to the *Tst* locus using the bioinformatics databases with their tools or programs as described below.

#### Ensembl genome database and tools

A 7400 bp-long segment of interest was analysed in the Ensembl database (Mouse genome coordinates:15:78399000 - 78406400) (release 79) [14]. Evolutionary constrained elements were extracted based on the nucleotide conservation between the 39 eutherian mammals in the “Location” tab under “Comparative Genomics – Alignments (text)” tree item. SNPs were categorized into variation consequence types – upstream gene variant, synonymous variant, intron variant, missense variant, nonsense variant, 5′ or 3′ untranslated region (UTR) variant and downstream gene variant. In the “Region in detail” tree item constrained elements for 39 eutherian mammals were obtained. DNA methylation state was indicated from dark blue (highly methylated), through green and then towards yellow (low methylation) and was based on the analysis in the mouse embryonic stem (ES) line. Data measuring chromatin activity or state (DNase I hypersensitive sites, histone modifications, polymerase II and III binding elements) and transcription factor binding sites were extracted and cell specific peaks were marked. Predicted miRNA targets from the Ensembl Regulation database were searched and displayed in the regulatory element map.

#### Eukaryotic promoter database (EPD)

Experimentally validated eukaryotic RNA polymerase II promoter and TATA-box for *Tst* gene were retrieved from the EPD (EPDnew Mouse version 002) [23].

#### AliBaba2.1

An upstream sequence of the *Tst* gene (15:78405000 - 78406400) was analysed for predicted transcription factor binding sites using Alibaba2.1 program [24]. Default parameters settings were employed.

#### MotifMap

The MotifMap [25] system was used to obtain additional predictions of candidate regulatory elements. In the “Gene search” window, *Tst* gene was selected and searched for transcription factor binding sites in regions upstream and downstream of its transcription start site (TSS). All filters were kept as default, except upstream distance from TSS was set to 500 bp.

#### CpG island prediction

UCSC Genome Browser [26] and three additional web tools MethPrimer [27], CpG Island Searcher [28] and EMBOSS CpGplot [29] were used for searching CpG islands within the 0.5 kb DNA sequence upstream of the *Tst* gene. The islands are predicted by searching the sequence one base at a time, scoring each dinucleotide and identifying maximally scoring segments. These segments are then evaluated for the criteria: GC content > 50, length > 200 bp, observed/expected CpG ratio > 0.6 [30].

#### MicroRNA miRNA target analysis

miRNA target prediction tools (miRWalk [31], miRDB [32], MicroCosm [33], miRecords [34]) were used to identify potential binding sites for miRNAs within the 3′UTR and promoter of the *Tst* gene that could potentially function in *Tst* mRNA translatability.

### Sequencing the *Tst* locus in F and L lines

Genomic DNA was isolated from spleen and liver of two mice per parental F and L line, using the procedures provided by the GenElute™ Mammalian Genomic DNA Miniprep Kit (Sigma). DNA concentration was determined using a NanoVue Spectrophotometer (GE Healthcare Life Sciences). The ~10 kb segment was divided in three overlapping smaller fragments (A, B, C; Additional file 11: Figure S2-A). PCR amplification was carried out using components from the Phusion High-Fidelity PCR Kit (Thermo SCIENTIFIC). The reaction mix contained 5x Phusion GC buffer, 10 mM dNTPs, 10 μM forward and reverse primers (Additional file 10: Table S10), 2 U/μl Phusion DNA Polymerase and 50 ng/μl of gDNA in a total reaction volume of 20 μl. Thermocycler conditions were 98°C for 30 s, followed by 35 cycles of 98°C for 7 s 68°C for 30 s and 72°C for 3 min and one cycle at 72°C for 10 min. Samples were verified on 1.5 % agarose gel and purified with GenElute™ PCR Clean-Up Kit (Sigma). Each amplified segment was then analysed in 10 μl sequencing reactions, containing 20–80 ng/μl of PCR product and 5 μM primers for detailed Sanger-sequencing (GATC Biotech). The MEGA 6 software [35] was used to assemble obtained sequences and to examine possible variations between the two mouse lines. Positions and direction of forward and reverse sequencing primers are displayed schematically in Figure S2-B (Additional file 11: Figure S2) whereas, nucleotide sequences of these primers are collated in Table S10 (Additional file 10: Table S10).

Ethics statement: DNA was isolated from mouse spleen and liver of two mice per parental F and L line at 14 weeks of age. Mice were humanely euthanised using CO_2_ followed by cervical dislocation. The use of animals to obtain mouse tissues for DNA isolation has been performed in accordance with the directive of the European Union 2010/63, approved by the Slovenian Ethical Committee for Animal Research and the Ministry of Agriculture, Forestry and Foods, Republic of Slovenia (licence No. U34401-55/2013/6).

## Results and discussion

Evaluating how genomic variations in candidate genes for complex traits could affect the function and, consequently, the phenotype is a necessary step in positional cloning projects. Even when such projects result in identification of a gene responsible for the phenotypic effect we are still faced with a task of identifying the gene variant that is causal. This process, called quantitative trait nucleotide (QTN) identification, still presents a daunting task and can be specific for a particular gene or trait under study [36]. What is common to such projects is development of a priority list of gene variants for a particular candidate gene. The priority variant list is then used for validation in functional studies. Our general approach to build this integrative map is detailed in Additional file 12: Figure S1. In the first part of the study we here developed a bioinformatics approach to integrate existing knowledge into a regulatory element map of the *Tst* locus. In the second part, the entire *Tst* locus was sequenced between our mouse strains to uncover genetic variation. We then used the regulatory element map to prioritise likely causal *Tst* gene variants in the F and L lines.

### Locus-wide analysis of the *Tst* regulatory elements

To construct an integrative map of important regulatory elements of the *Tst* locus, we employed various bioinformatics tools and databases as shown in the schematic overview in Additional file 12: Figure S1. Specifically, we searched for chromatin structure-related features such as histone modifications, open chromatin regions, potential DNA methylation sites, transcription factor, RNA polymerase and miRNA binding sites, as well as for genetic variants in all known sequenced mouse strains. As we used multiple available bioinformatics tools that often report or identify different elements or different size ranges for the same element, we decided to include all available information in a comprehensive map of *Tst* regulatory elements (Fig. 1).

The top panel of Fig. 1 shows the gene structure of the *Tst* gene. Ensembl database-derived reference sequence of the mouse *Tst* gene is 7400 bp-long and consists of 2 exons and one intron. The Ensembl or the UCSC Genome Browser do not provide annotations of any other overlapping protein or non-coding RNA gene in this segment, which was confirmed by our comprehensive bioinformatics analyses using other tools and prediction programs (see below). We next searched for evolutionary conservation of the ~ 7.4kb segment across a set of 39 eutherian mammals to identify constrained elements (Fig. 1a). Other regulatory features (Fig. 1b-h) overlapping with sequences of constrained elements are emphasized with a grey area in Fig. 1a. These constrained elements can serve as a guide to pinpoint regions of noncoding or coding DNA with conserved biological functions, and thus may be more likely to harbour SNP variants with functional consequences [37]. A total of 12 stretches of highest sequence conservation were identified amongst which the longest two regions covered the two exons, as expected. Nine of 12 highly conserved regions were identified also in the non-exonic sequences, eight in the intron and one in the flanking sequence upstream of the *Tst* promotor. However, our result of eight conserved blocks presenting 5 % of intronic sequence suggests that the *Tst* intron contains fewer highly conserved elements compared with 12–28 % found in three mammalian orders [38].

The formation of open chromatin in eukaryotic genomes is an important factor controlling potential regulatory activity [39]. Such active functional elements can be located through the identification of regions of the genome that are hypersensitive to DNase I cleavage [40], so called DNase I hypersensitive sites or regions of open chromatin. The ENCODE project uncovered two open chromatin sites for the mouse *Tst* gene (Fig. 1b). The proximal DNase I hypersensitive site was found within the intron. A larger DNAse I hypersensitive site was located distally and overlapped the *Tst* promoter. The existence of functional open chromatin sites is corroborated by mapping TF binding sites within these regions using two different transcription factor binding sites (TFBS) analyses algorithms (Alibaba2.1 and Motifmap) and Ensembl database annotations (Fig. 1c; Additional file 7: Table S7). Additionally, Ensembl database annotated various histone modification regions (Fig. 1d) to the two aforementioned open chromatin regions. RNA polymerase II binding sites have been experimentally demonstrated in two different cell lines examined by ENCODE within the promoter open chromatin region (Fig. 1e). Therefore, several lines of evidence support the existence of two *Tst* open chromatin regions with a regulatory role, a smaller intronic region and a larger promoter open chromatin region.

DNA methylation marks potential sites for epigenetic regulation found to be important in many biological processes especially in gene expression regulation. We found 118 CpG sites in the examined 7.4 kb *Tst* genome segment. Cytosine methylation varied from 0 % (unmethylated) to 100 % (methylated), which is displayed (Fig. 1f) as height bars. Methylation of large CG-rich stretches of DNA especially in promoters strongly correlates with suppression of gene expression [41]. We used various tools to identify CpG islands (Fig. 1g) which all predicted two clusters of non-methylated CpG islands. This segment co-localized with the predicted promoter, indicating its potential role in transcriptional regulation of *Tst*. Micro RNA miRNA binding site prediction tools (Fig. 1h) provide strong evidence for the existence of miRNA regulatory sites within the 3′UTR located in the second *Tst* exon (Fig. 1h).

**Fig. 2:**
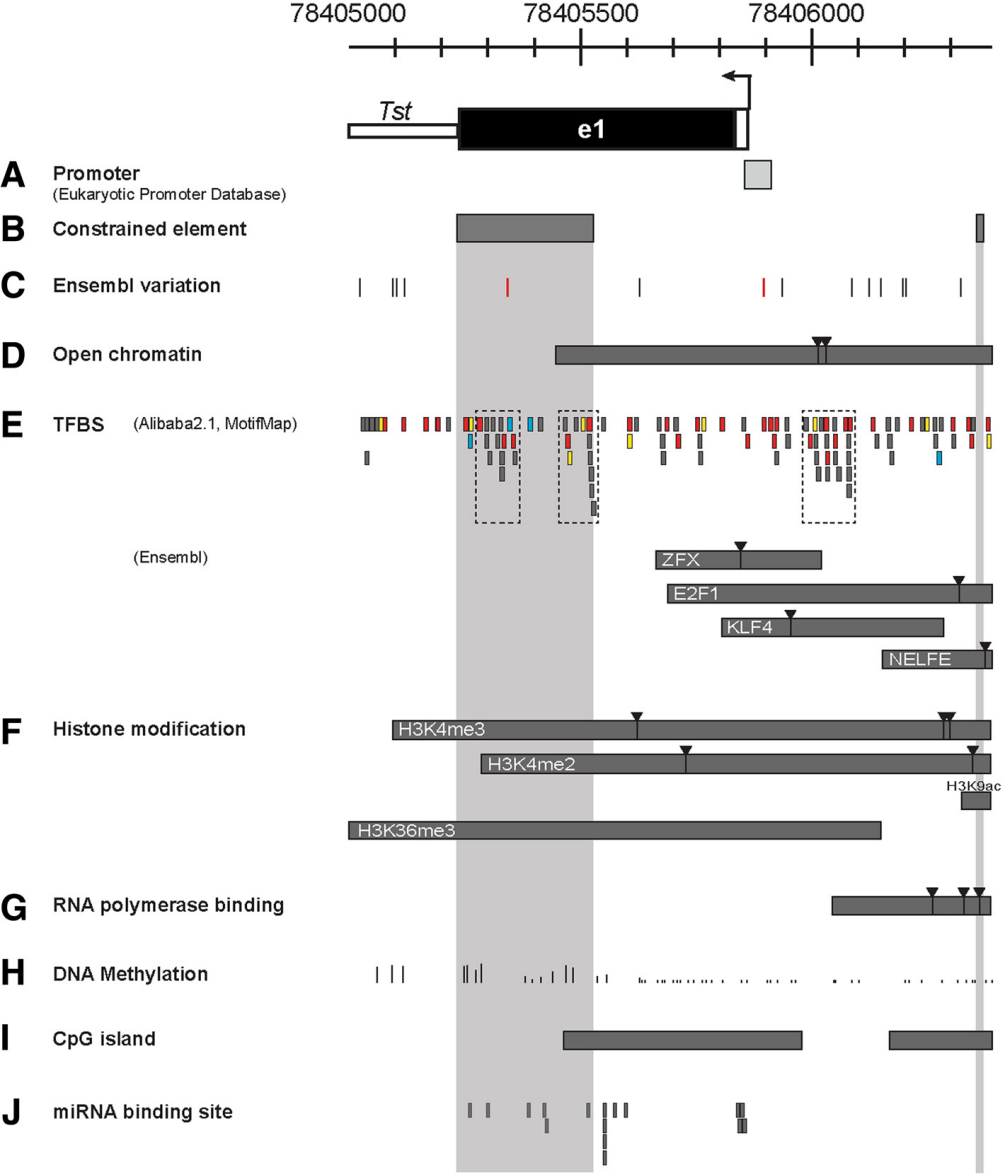
Regulatory conserved region of mouse *Tst* gene – *Tst* core promoter and exon **1**. Black arrow above exon 1 presents transcription start site (TSS). **a** Grey box presents promoter region of *Tst* gene. **b** Grey boxes show constrained elements between 39 eutherian mammals. **c** Lines presenting conserved polymorphism. **d** DNase I binding presented as grey box with its peaks as inverted triangles. **e** Thick grey lines showing predictions of TF binding sites. Red lines present binding site for SP1, yellow lines present binding site for NF1 and blue lines present binding site for C/EBPα transcription factor. Grey stripes present ENCODE data with peaks (inverted triangle) for TFBS (**e**), histone modification (**f**) and RNA polymerase binding (**g**). **h** The degree of DNA methylation is presented as height line (100 % (highest line) – 0 % (lowest line) methylation). **i** Two CpG island stripes cover the predicted CpG islands from various prediction tools. **j** Thick grey lines display predicted miRNA target sites within *Tst* exon 1. *Zfx* – Zinc finger protein X-linked; *E2F1* – E2F transcription factor 1; *Klf4* – Kruppel-like factor 4 (gut); H3K4me3 – Histone 3 lysine 4 trimethylation; H3K4me2 – Histone 3 lysine 4 dimethylation; H3K36me3 – Histone 3 lysine 36 trimethylation

**Fig. 3:**
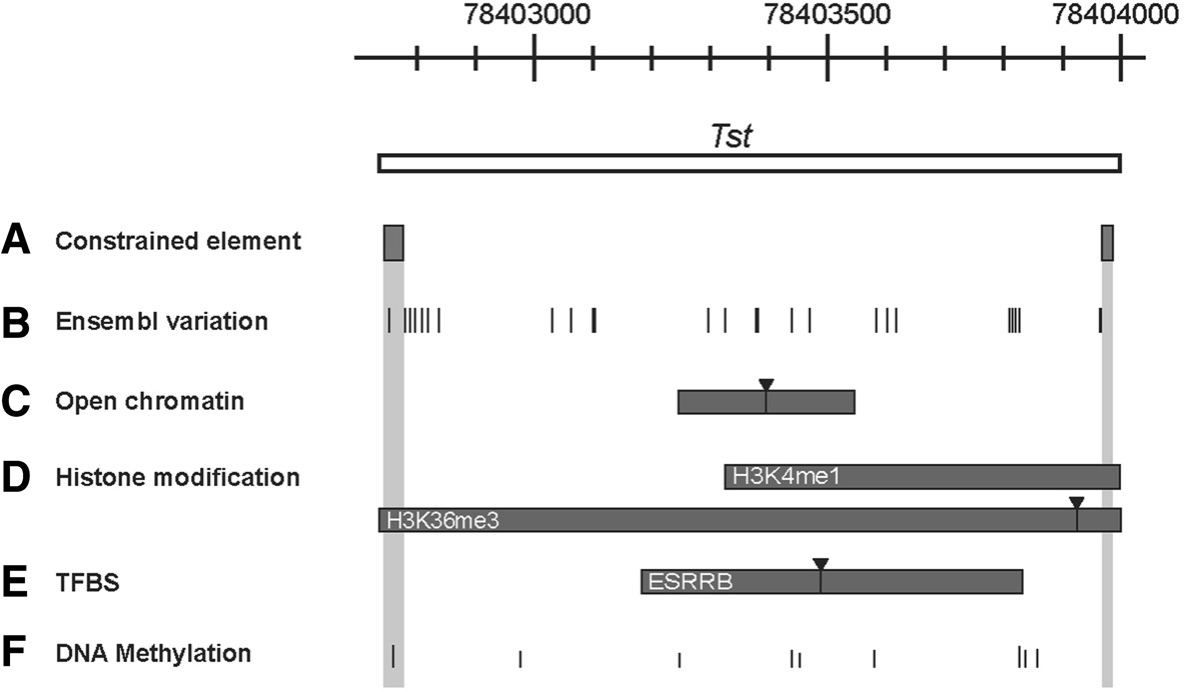
Regulatory conserved region of mouse *Tst* gene – intron. **a** grey boxes show constrained elemnts between 39 Eutherian mammals. **b** Lines present conserved polymorphisms. Stripes present data froom ENCODE project with peaks (inverted triangles) for (**c**) open chromatin, (**d**) histone modifications and (**e**) TFBS. **f **The degree of DNA methylation is presented as a height line (100% methylation is the highest line, while 0% methylation is the lowest line). H3K4me1 – Histone 3 lysine 4 methylation; H3K36me3 – Histone 3 lysine 36 trimethylation; Esrrb – Estrogen related receptor beta

**Fig. 4:**
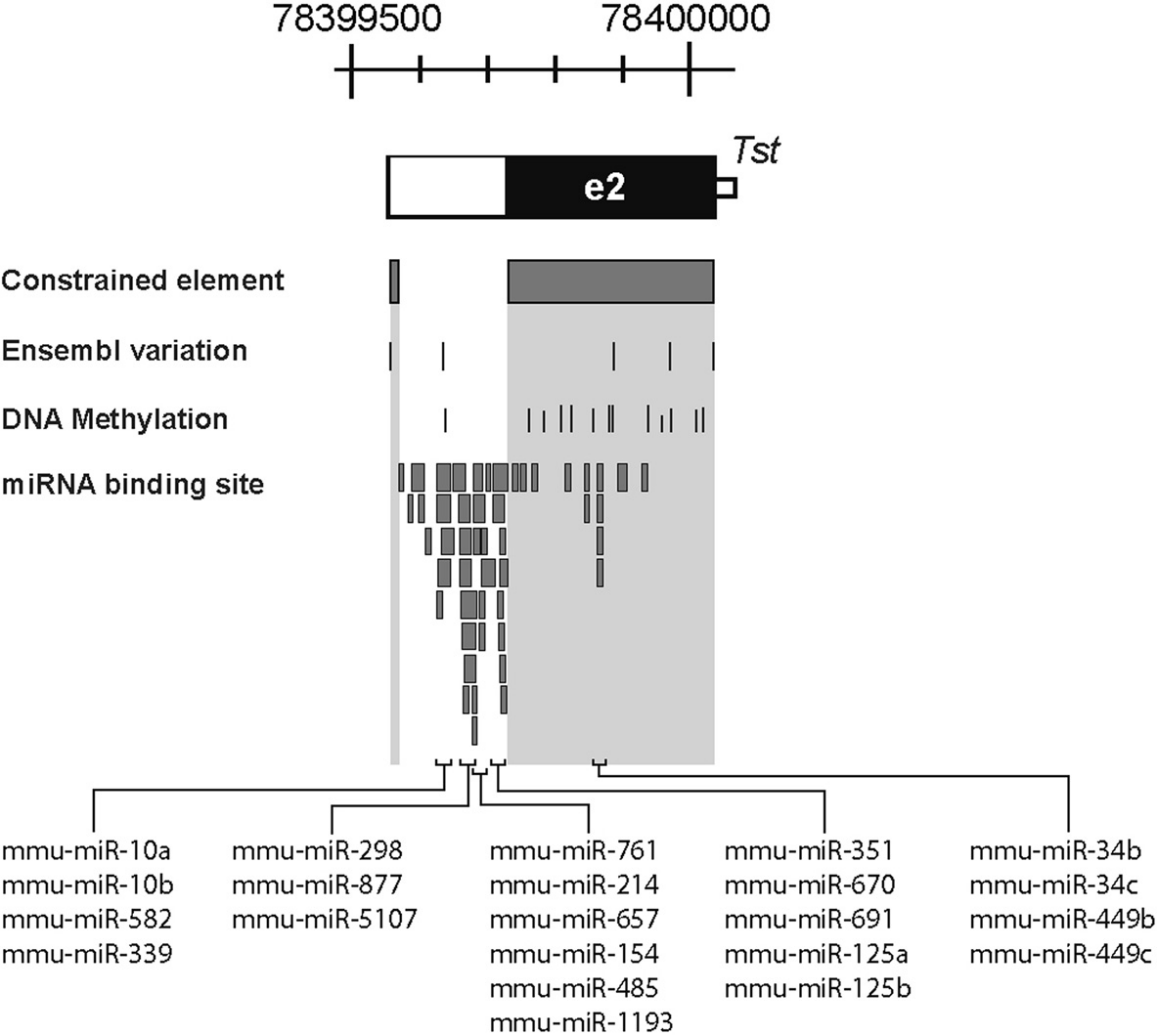
Regulatory conserved region of mouse *Tst* gene – exon 2. Thick bars show miRNA predicted target sites on *Tst* 3′UTR and exon region

Therefore, a comparative analysis of various regulatory features provides strong evidence for existence of three regulatory genome segments. These locate to the promoter, intron and 3′UTR of the *Tst* locus (marked at the bottom of Fig. 1) and will be explored in more detail in the following sections and Figs. 2, 3 and 4).

### Regulatory features of the *Tst* core promoter and exon 1 region

To define the location of the core promoter sequences we used the Eukaryotic promoter database (EPD) tools that located it within a 59 bp-long interval overlapping the transcription initiation site (TSS, Fig. 2a – black). No TATA-box or CG box consensus sequences were identified around this core promoter segment. Genetic variation in the mouse genome in this segment identified 14 SNPs, one of which mapped to the evolutionary constrained element (Fig. 2b) and one SNP to the core promoter (Fig. 2c, presented as red lines). SNPs occurring in highly conserved functional regions may affect expression and phenotypic variability in strains harbouring the mutant alleles. The open chromatin region (Fig. 2d) harbours four transcription factor binding sites (TFBS) for transcription factors ZFX, E2F1, KLF4 and NELFE (Fig. 2e). Ensembl annotates these TFBS from ChIP-Seq experiments in selected cell lines (ES and MEL) so it is possible that some TFs are missed as they don’t regulate TST in the examined cell lines and/ or act *in vivo* in a tissue specific manner. For this reason we applied two additional tools, Alibaba2.1 and MotifMap, that predicted a variety of different transcription factors (Fig. 2) potential binding sites (*n* = 55) in the region of open chromatin (Fig. 2d-e; Additional file 7: Table S7) as well as constrained element (Fig. 2b). One has to be cautious about all these predicted TFBS as they are not experimentally verified and do not tell us anything about tissue specificity. However, the SP1 binding site was frequently predicted (Fig. 2e, red thick line) and this TF has already been linked as a cellular glucose sensor and it is also involved in leptin promoter activity [42]. In line with this is our observation that SP1 sites overlap with open chromatin region. In addition, Alibaba and MotifMap algorithms frequently predicted sites for NF1 (Fig. 2e, yellow thick line), a transcription activator binding protein [43] and C/EBPα (Fig. 2e, blue thick line), a liver-enriched transcriptional regulator involved in energy metabolism [44]. In the context of *Tst* regulation, none of these sites have actually been experimentally validated. However, TFBS are clustered in three small segments (dashed box in Fig. 2e), two within the proximal constrained element and the third on top of the peak of open chromatin site (between 78406000 - 78406100). Additionally, TFBS overlap with the less methylated CpG sites (Figs. H, I), open chromatin, RNA polymerase binding sites and histone modification motifs specific for active transcription. Therefore, our analysis of TFBS offers grounds and directions for further experimental work to examine which TFBS are functional for tissue-specific *Tst* regulation.

Three different histone modifications (Fig. 2h) were identified in this segment: H3K4me3 - one of the most studied chromatin modifications, present at actively transcribed protein coding promoters in eukaryotes; H3K4me2 – these modifications are enriched within TFBS sites [45]; and H3K36me3 – enrichment of these modifications was found to be higher at exonic than intronic regulatory sites within the *Tst* locus, which is in line with previous reports [46]. We conclude that the *Tst* core promoter, 5′UTR and exon 1 region contain most types of histone modifications typically found in promoters of actively expressed protein coding genes.

### Regulatory features of the *Tst* intronic region

Our bioinformatics analysis revealed strong evidence for the existence of the second regulatory region within intron of the *Tst* gene (Fig. 1). Introns can affect transcription by acting as repositories for transcriptional regulatory elements (i.e. enhancers and repressors). We narrowed a segment potentially important in the regulation of *Tst* to a region between 78402750 – 7840000 bp. Using different tools and databases we identified regulatory elements or features such as constrained elements (Fig. 3a), open chromatin site (Fig. 3c), two histone modifications specific for transcribed portions of genes and associated with enhancers and other distal elements (Fig. 3d), and also one TFBS (Fig. 3e). Additionally, the frequency of DNA methylation sites (Fig. 3f) was statistically significantly reduced in this segment when compared with the frequency of DNA methylation in the rest of the intron (Chi-square test, p-value < 0.01). Overlapping regulatory intronic features such as open chromatin, histone modification H3K4me1 and the binding site for transcription factor estrogen related receptor, beta (ESRRB) could indicate the presence of a functional intragenic enhancer for *Tst* or flanking genes, as was shown for some other genes [47]. Direct involvement of ESRRB in the regulation of *Tst* gene has not yet been demonstrated. However, binding of ESRRB to a consensus TFBS as the one found in intron of *Tst*, was experimentally mapped in the mouse ES cells which also supported previous genetic studies [48–50] suggesting its role in the self-renewal of ES cells. Of note, for the context of our study and the Fat and Lean model of mice, is the study of Li et al. [51] that achieved full reprogramming of naıve-like porcine adipose induced stem cells and linked this process, in part, by significant up-regulation of ESRRB. Transcriptomics studies also determined RNA expression of *Esrrb* in adipocytes [52]. Therefore, our results suggest that ESRRB could be regulating abundance of TST protein in a tissue (adipose) specific manner by using the identified binding –enhancer site in intron of *Tst*.

### Regulatory features of the *Tst* 3′UTR region

We positioned the third important regulatory region in the second exon of the *Tst* gene (Fig. 4). Approximately half of the distal part of this exon contains the 3′UTR region that is frequently found to regulate gene expression at the post-transcriptional level in eukaryotes [53]. Regions conserved and important for such regulation are around a poly(A) tail consensus site (5′-AATAAA-3′), found between 78399577 – 78399572 bp which in cooperation with poly(A) binding proteins, contributes to regulation of mRNA translation, stability and export. It could also be possible that the structural characteristics of the 3′UTR may contribute to gene expression, as in general, longer 3′UTR correlate with lower expression rates, since they contain more miRNA and protein binding sites that are involved in inhibiting translation. We searched for miRNA binding sites using various prediction tools such as miRWalk [31], miRDB [32], MicroCosm [33], and miRecords [34]. As shown in Fig. 4 there is a considerable overlap of hits by these various tools providing strong support for existence of miRNA sites in the 3′UTR of *Tst*. Two clusters emerged, one in the coding sequence of exon 2 and a more prominent miRNA binding region in the 3′UTR. A total of 54 different miRNA were predicted to bind four sites within 3′UTR, among which mmu-miR-10a, mmu-miR-10b, mmu-miR-761, mmu-miR-214, mmu-miR-670, mmu-miR-877 and mmu-miR-339 were predicted by almost all used tools. That mammalian 3′UTR regions can be targeted by multiple miRNAs has recently been demonstrated in a large-scale genomic screen of miRNA-mRNA interactions [54]. That the predicted four miRNA binding sites in the 3′UTR of *Tst* may indeed be functional is supported by reduced genetic variation in these sites among 15 mouse strains. Only one miRNA binding site was found to be variable with a non-reference nucleotide found only in 4 out of 15 strains as will be explained in more detail in the next section. Furthermore, the location and structural features of the four sites indicate that the sites may indeed be functional and efficient. Sites are positioned at least 15 nucleotides downstream of the stop codon and are close to one end of 3′UTR, which are both strong features of effective miRNA binding sites as found in an experimental study of Grimson et al. [55]. The four sites also exhibit AU‑richness and lie in unstructured areas, which were experimentally found to correlate with increased accessibility for the miRNA regulatory complex [56]. Therefore, on the basis of our bioinformatics analysis that identified several potential miRNA binding sites whose target site location and sequence context imply functional efficacy. We can conclude that the *Tst* locus is likely to be rich in miRNA target sites and hence likely to be regulated by miRNAs to affect expression or tissue specificity.

### Identifying and prioritising polymorphisms of the Fat and Lean lines using the regulatory element *Tst* map

#### Sequence analysis of *Tst* locus in F and L lines

**Fig. 5:**
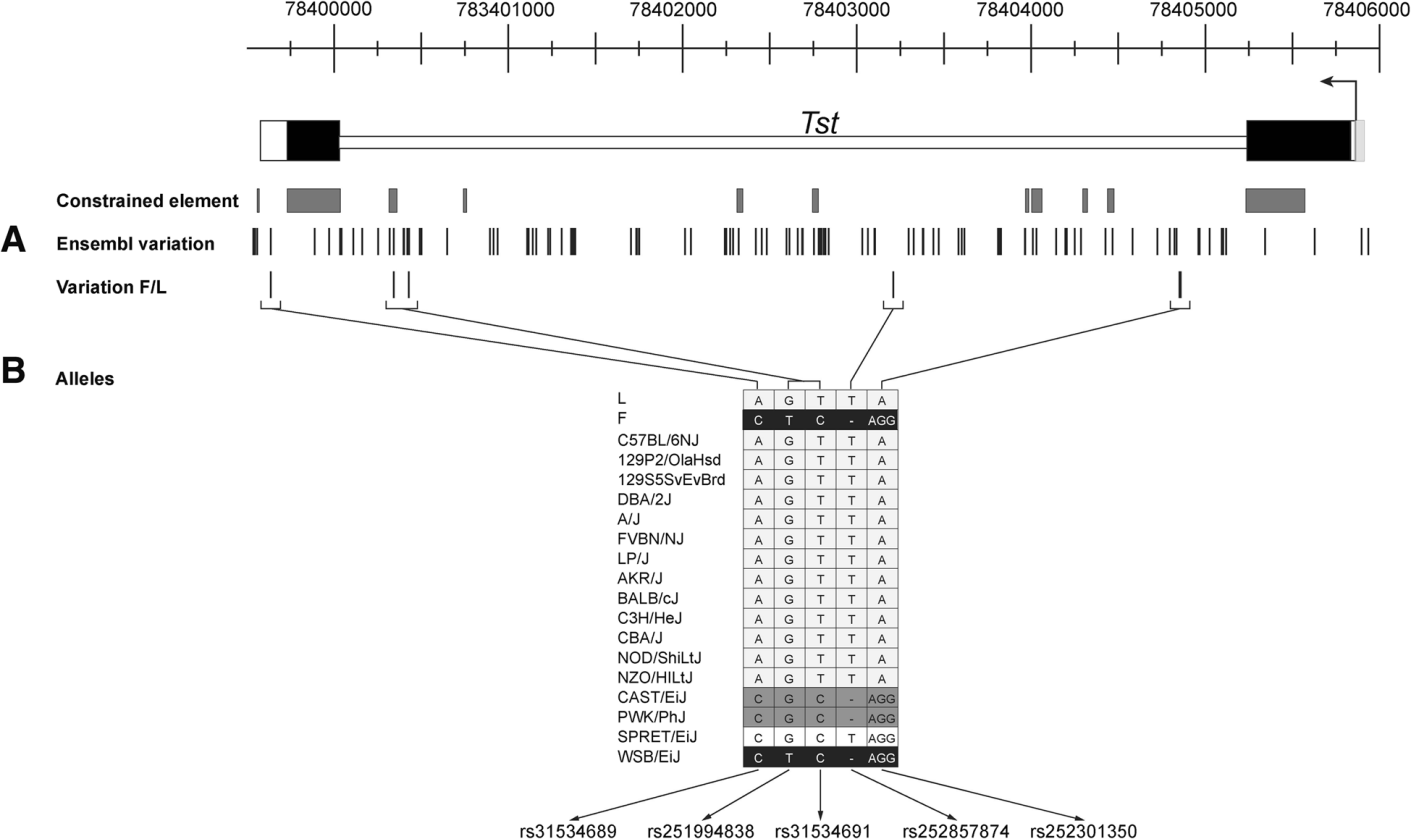
Identified variations between Fat and Lean mouse line within the *Tst* region. **a** Three SNPs and two indels were determined between F and L lines. **b** Allele characterization of 17 most-used strains of mouse (The genetic sequence variations of 17 common laboratory mouse strains)

Once the integrative map of potentially functional regulatory elements within the *Tst* locus was constructed (Figs. 1, 2, 3 and 4 above), we used it for prioritising genetic variants potentially causal for the *Tst* in our obesity/leanness mouse lines. To accomplish this, we first needed a high resolution sequence of the *Tst* locus in both lines to identify all possible polymorphisms. The sequencing strategy and locus view of 10962 bp-long sequenced segment (Chr 15: 78399030 - 78409992 bp) covering the *Tst* gene plus additional 4133 bp sequence of the promoter (Chr15: 78405859 - 78409992) and 526 bp downstream of the 3′ end of the gene (Chr15: 78399556 - 78399030) is shown in Additional file 11: Figure S2. The aligned sequences between the F and L mouse lines revealed only five polymorphisms (Fig. 5a). Considering the length of the mouse genome and a total number of short variants one would expect on average 39.4 bp per variant. With only five variants between the Fat and Lean line this rate is significantly reduced to about one variant per 1050 bp indicating that this genomic region in F and L mice is likely derived from two closely related progenitor lines. In comparison with the reference sequence of the mouse genome (strain C57BL/6J) the Fat and Lean lines contained 4 SNPs, a one nucleotide deletion and a dinucleotide insertion in the Fat line (Fig. 5b). Apart from SNP rs31534689 that resides in the 3′UTR of the second *Tst* exon and rs251994838 located in a constrained element, all other gene variants mapped to the intron whereas none were identified in ~ 5kb segment upstream of transcription start site. The sequence analysis of the Fat and Lean lines revealed that no private alleles, i.e. specific to Fat or Lean line only, exist, which is in line with previous data demonstrating that only ~2 % private variants exist across the whole genomes of laboratory strains of mice [57]. Sequencing of the 10962 bp-long segment covering the *Tst* locus in the F and L lines identified five polymorphisms providing a basis for further comparative genomic analyses and prioritisation of potential functional variants.

#### Strain of origin of the *Tst* region in the F and L lines

Once we had a complete and high resolution sequence of the *Tst* locus from the Fat and Lean lines we asked if we can identify from which mouse strain, species or subspecies is this locus derived from in Fat and Lean mice. As alluded in the introduction, Fat and Lean lines were selected for more than 60 generations for high or low body fat % from a common base population derived from crosses of two inbred lines JU and CBA and an outbred strain CFLP from the Carnworth laboratory [15]. As none of the samples from these original base population strains are available, it is not possible to definitively ascertain the origin of the *Tst* locus DNA in our current Fat and Lean inbred lines. However, on the basis of our new sequence data we can predict the likely origin from comparative sequence and haplotype analysis. The Fat line shared the exact haplotype of all gene variants only with the WSB/EiJ strain but was also very similar (four of five variants in common) with other wild-derived mouse strains such as CAST/EiJ, PWK/PhJ and SPRET/EiJ. Since the wild derived WSB/EiJ strain is a representative of *Mus musculus domesticus* subspecies [57] we conclude that the Fat line also contains the *M. m. domesticus* DNA at the *Tst* locus. The Lean line shared a haplotype for the five variants with 13 other classical inbred mouse strains including the reference C57BL/6J strain (Fig. 5b). As demonstrated before [58], these classical laboratory strain genomes are mosaics of genomes from four taxa, *Mus musculus castaneus*, *Mus musculus musculus*, *Mus musculus domesticus* and *Mus spretus*. The mouse genome, in contrast to human, where projects like 1000-human genomes have already been accomplished, lacks comprehensive data on variation of natural mouse populations from which the classical laboratory strains were derived [59]. It is thus not possible to assign local ancestry at a finer scale and this is the case for the *Tst* locus in the Lean line. However, our sequencing and haplotype analyses results do suggest that the Fat line *Tst* locus has a “wild type” haplotype and the Lean line allele is of the inbred laboratory strain origin. This is in line with our previous transcriptomics results demonstrating overexpression of *Tst* in the Lean line over the comparator (“wild type” *Tst*) expression of the Fat line [22]. It is likely that one, or a combination of the five identified genetic variants are responsible for this overexpression effect.

#### Prioritisation of genetic variants in relation to the *Tst* regulatory element map

As our final goal is to identify the causal genetic variant for the phenotypic effect on leanness/obesity of the *Tst* locus it is important to prioritise potential candidate genetic variants before embarking upon more focused and lengthy functional analyses downstream. As the majority of genetic variants in genome-wide association studies in humans and animal models as well as re-sequencing projects are located in non-coding regions, this hinders the obvious assessment of their functional effect as the protein sequence itself is not modified.

Here we combined our bioinformatics-based regulatory atlas of the *Tst* locus and identified genetic variants between our target strains to prioritise them functionally and hence improve our chances to select potential causal variants for further experimental work.

**Fig. 6:**
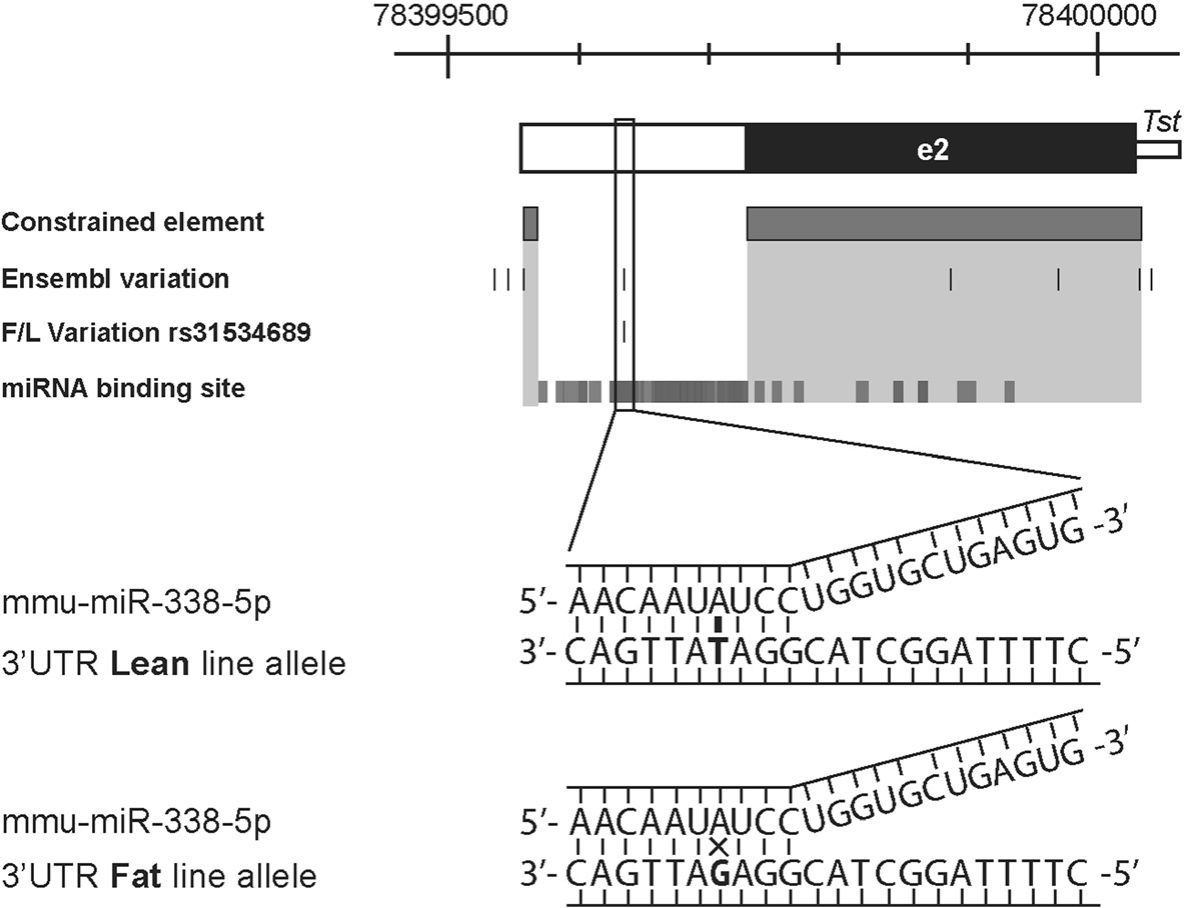
*Tst* 3′UTR variation within predicted target site for miRNA mmu-miR-338-5p. Cross-section of predicted miRNAs and their targeting *Tst* sites with sequenced polymorphisms, revealed one miRNA that fully bind with its seed sequence to the lean line allele in 3′UTR SNP. Thick bars show predicted miRNA target sites on *Tst* 3′UTR and exon region

Non-coding SNPs can have large cis-regulatory effects if they lie in important regulatory DNA motifs *via*, for example, altering their affinity for TFs, splicing procedures or chromatin remodelling processes. In our case, only one intronic variant, rs251994838, overlapped with an evolutionary constrained intronic element and could hence have a regulatory impact on the *Tst* gene expression by affecting DNA accessibility (chromatin structure) or the affinity of transcription factors binding. The second high priority genetic variant candidate (rs31534689) lies in the 3′UTR (Fig. 6). Our bioinformatics analysis identified a large cluster of potential miRNA binding sites around this genetic variant using various miRNA binding site prediction tools. Amongst potential miRNA species, miRNA mmu-miR-338-5p is predicted to bind to the 3′UTR Lean line allele with no mismatches and with a mismatch to the Fat line allele (Fig. 6). The rs31534689 polymorphism is located in a so-called seed region of miRNA [60], where complete complementarity plays a major role in miRNA target recognition. This SNP therefore has a potential to affect differential *Tst* mRNA stability or translatability. We conclude that rs251994838 and rs31534689 represent the highest priority candidate genetic variants for further experimental functional validation of their causality on the phenotype. As *Tst* was found to be overexpressed in the Lean line specifically in the white adipose tissue [61], such validations will likely have to be carried out in the context of adipocyte cell lines or white adipose tissue. New CRISPR-based transgenesis approaches also offer promising tools to evaluate causality by allele replacement approaches *in vivo*.

## Conclusions

In our recent positional cloning experiment we identified *Tst* as the causal gene for the *Fob3b2* QTL phenotypic effect on leanness with improved metabolic parameters [22]. As increased *Tst* expression selectively in adipose tissue was found responsible for this effect, we focused here on developing a map of regulatory elements for the *Tst* locus in mice potentially involved in regulating the mRNA transcript levels encoding this enzyme. The map provides a basis for planning further experimental validations and functional analyses of this important and evolutionary conserved gene. It helped to narrow down *Tst* genetic variants to a small testable list of candidate polymorphisms for more focused and hypothesis-driven experimental work. Our approach combined information on evolutionary constrained elements, promoter, enhancer, epigenetic and chromatin-related regulatory features, protein and ncRNA binding sites and genetic variants into a single integrated regulatory element map. This approach, demonstrated here for the *Tst* locus, is of general utility and could be used to develop regulatory element maps for other genes also in other species to classify, prioritize and functionally interpret potentially causal regulatory genetic variation. As most complex trait and disease associations detected by genetic studies lie outside coding regions, developing a regulatory element map in candidate genes could inform which annotations of gene structures and regulatory elements contain likely causal variants in the target gene under study. This can lead to identification of regulatory variants and interpretation of how variation in regulatory mechanisms results in controlling gene expression or activity. A map of regulatory elements described in this study can therefore provide additional layer of biologically-relevant information to the genome sequence itself and serve as the basis for post-GWAS or post-QTL functional studies.

## Abbreviations

F, fat line; gDNA, genomic DNA; GWAS, genome-wide association study; L, lean line; miRNA, micro RNA; qPCR, quantitative polymerase chain reaction; QTL, quantitative trait loci; RT, reverse transcription; SNPs, single nucleotides polymorphisms; *Tst*, thiosulfate sulfurtransferase

## Additional files

Additional file 1: Table S1. Constrained elements for 39 eutherian mammals (Ensembl). (DOCX 15 kb) Additional file 2: Table S2. Sites enriched for marks of open chromatin (Ensembl). (DOCX 15 kb) Additional file 3: Table S3. RNA polymerase binding sites (Ensembl). (DOCX 15 kb) Additional file 4: Table S4. CpG islands – genome coordinates. (DOCX 15 kb) Additional file 5: Table S5. Histone modifications (Ensembl). (DOCX 16 kb) Additional file 6: Table S6. DNA methylation in Embryonic stem cell. (DOCX 19 kb) Additional file 7: Table S7. Sites enriched for marks of transcription factor binding sites. (DOCX 23 kb) Additional file 8: Table S8. Predicted miRNA target sites. (DOCX 22 kb) Additional file 9: Table S9. Merged mouse single nucleotide polymorphisms (SNP) from Ensembl and Mouse genomes project (Wellcome Trust Sanger Institute). (DOCX 63 kb) Additional file 10: Table S10. Sequences of primers used in sequencing of *Tst* locus (region). (DOCX 17 kb) Additional file 11: Figure S2. Primers positions used in sequencing the *Tst* locus. A) Positions of primers used for amplification of ~ 3 kb segments (A, B, C) of *Tst* region. B) Location of PCR primers for detailed sequencing of A, B, and C segments. (JPG 4083 kb) Additional file 12: Figure S1. Integrated gene transcription regulatory elements for atlas development. Schematic of elements affecting gene transcription and expression at the level of chromatin state (histone modifications, DNA methylation, chromatin accessibility), through transcription factors and RNA polymerase binding, variation impact and microRNAs miRNAs influence. (JPG 1035 kb)
