# Supplementary material for: Construction of an integrative regulatory element and variation map of the murine Tst locus
Source: BMC Genet. 2016 Jun 11;17:77. doi: 10.1186/s12863-016-0381-6 (PMC4902921; doi:10.1186/s12863-016-0381-6)
Supplement: Additional file 1: Table S1. — Constrained elements for 39 eutherian mammals (Ensembl). (DOCX 15 kb) [file 12863_2016_381_MOESM1_ESM.docx]

Table S1. Constrained elements for 39 eutherian mammals (Ensembl).

| Chr: bp |
| --- |
| 15:78399558-78399569 |
| 15:78399731-78400035 |
| 15:78400316-78400360 |
| 15:78400742-78400760 |
| 15:78402312-78402345 |
| 15:78402745-78402778 |
| 15:78403968-78403986 |
| 15:78404004-78404062 |
| 15:78404296-78404322 |
| 15:78404438-78404474 |
| 15:78405233-78405571 |
| 15:78406355-78406370 |
