# Supplementary material for: Construction of an integrative regulatory element and variation map of the murine Tst locus
Source: BMC Genet. 2016 Jun 11;17:77. doi: 10.1186/s12863-016-0381-6 (PMC4902921; doi:10.1186/s12863-016-0381-6)
Supplement: Additional file 2: Table S2. — Sites enriched for marks of open chromatin (Ensembl). (DOCX 15 kb) [file 12863_2016_381_MOESM2_ESM.docx]

Table S2. Sites enriched for marks of open chromatin (Ensembl).

| Chr:bp | Peak summit | Cell type |
| --- | --- | --- |
| 15:78403247-78403547 | 78403396 | ES |
| 15:78405676-78406252 | 78406030 | ES |
| 15:78406387-78406996 | 78406841 | ES |
| 15:78405677-78406979 | 78406013 | ES |
| 15:78405447-78407029 | 78406565 | MEL |
