# Supplementary material for: Construction of an integrative regulatory element and variation map of the murine Tst locus
Source: BMC Genet. 2016 Jun 11;17:77. doi: 10.1186/s12863-016-0381-6 (PMC4902921; doi:10.1186/s12863-016-0381-6)
Supplement: Additional file 3: Table S3. — RNA polymerase binding sites (Ensembl). (DOCX 15 kb) [file 12863_2016_381_MOESM3_ESM.docx]

Table S3. RNA polymerase binding sites (Ensembl).

| Chr:bp | Peak summit | Cell type |
| --- | --- | --- |
| 15:78406180-78407052 | 78406328 | MEL |
| 15:78406198-78406543 | 78406362 | MEL |
| 15:78406044-78406505 | 78406264 | NPC |
