# Supplementary material for: Construction of an integrative regulatory element and variation map of the murine Tst locus
Source: BMC Genet. 2016 Jun 11;17:77. doi: 10.1186/s12863-016-0381-6 (PMC4902921; doi:10.1186/s12863-016-0381-6)
Supplement: Additional file 4: Table S4. — CpG islands – genome coordinates. (DOCX 15 kb) [file 12863_2016_381_MOESM4_ESM.docx]

Table S4. CpG islands.

| Chr:bp | Web tool and database |
| --- | --- |
| 15:78405479-78405979 | CpG Island Searcher |
| 15:78406168-78407043 | CpG Island Searcher |
| 15:78405463-78405898 | MethPrimer |
| 15:78406392-78406772 | MethPrimer |
| 15:78405613-78405890 | EMBOSS Cpgplot |
| 15:78405627-78405862 | UCSC |
| 15:78406316-78406690 | UCSC |

Criteria: lenght > 200 bp; CG % > 50; obs/exp CG % > 60.
