# Supplementary material for: Construction of an integrative regulatory element and variation map of the murine Tst locus
Source: BMC Genet. 2016 Jun 11;17:77. doi: 10.1186/s12863-016-0381-6 (PMC4902921; doi:10.1186/s12863-016-0381-6)
Supplement: Additional file 5: Table S5. — Histone modifications (Ensembl). (DOCX 16 kb) [file 12863_2016_381_MOESM5_ESM.docx]

Table S5. Histone modifications (Ensembl).

| Modification | Chr:bp | Peak summit | Cell type |
| --- | --- | --- | --- |
| H3K4me3 | 15:78405094-78407369 | 78406298 | ES |
|  | 15:78405174-78407060 | 78406298 | ES |
|  | 15:78405385-78406728 | 78405622 | ESHyb |
|  | 15:78405414-78407212 | 78406874 | MEL |
|  | 15:78405853-78407310 | 78406285 | MEF |
|  | 15:78406034-78406993 | 78406839 | NPC |
| H3K4me2 | 15:78405285-78406163 | 78405728 | ES |
|  | 15:78406170-78407125 | 78406348 | ES |
| H3K4me1 | 15:78403326-78404273 | 78404052 | MEL |
| H3K9ac | 15:78406323-78407520 | 78406610 | MEF |
| H3K36me3 | 15:78401550-78406150 | 78403925 | MEL |
