# Supplementary material for: Construction of an integrative regulatory element and variation map of the murine Tst locus
Source: BMC Genet. 2016 Jun 11;17:77. doi: 10.1186/s12863-016-0381-6 (PMC4902921; doi:10.1186/s12863-016-0381-6)
Supplement: Additional file 6: Table S6. — DNA methylation in Embryonic stem cell. (DOCX 19 kb) [file 12863_2016_381_MOESM6_ESM.docx]

| Table S6. DNA methylation in Embryonic stem cell (Merged replicates from Whole Genome Bisulphite Sequencing; Ensembl) |
| --- |

| Chr: bp | Methylated reads |
| --- | --- |
| 15:78399029 | 12/13 (92%) |
| 15:78399310 | 19/21 (90%) |
| 15:78399398 | 11/12 (91%) |
| 15:78399476 | 5/6 (83%) |
| 15:78399535 | 6/7 (85%) |
| 15:78399639 | 11/13 (84%) |
| 15:78399763 | 10/13 (76%) |
| 15:78399784 | 15/15 (100%) |
| 15:78399809 | 16/16 (100%) |
| 15:78399824 | 13/15 (86%) |
| 15:78399857 | 10/10 (100%) |
| 15:78399880 | 10/10 (100%) |
| 15:78399885 | 10/10 (100%) |
| 15:78399937 | 8/13 (61%) |
| 15:78399959 | 14/16 (87%) |
| 15:78399972 | 13/16 (81%) |
| 15:78400009 | 13/14 (92%) |
| 15:78400020 | 14/14 (100%) |
| 15:78400161 | 14/15 (93%) |
| 15:78400194 | 16/18 (88%) |
| 15:78400398 | 20/23 (87%) |
| 15:78400449 | 14/16 (87%) |
| 15:78400486 | 14/15 (93%) |
| 15:78400524 | 11/16 (68%) |
| 15:78400618 | 12/14 (85%) |
| 15:78400694 | 3/4 (75%) |
| 15:78400815 | 12/13 (92%) |
| 15:78400877 | 13/13 (100%) |
| 15:78400881 | 11/11 (100%) |
| 15:78400963 | 15/18 (83%) |
| 15:78401109 | 14/14 (100%) |
| 15:78401160 | 15/16 (93%) |
| 15:78401240 | 8/8 (100%) |
| 15:78401247 | 7/8 (87%) |
| 15:78401372 | 5/6 (83%) |
| 15:78401380 | 6/6 (100%) |
| 15:78401380 | 6/6 (100%) |
| 15:78401505 | 17/19 (89%) |
| 15:78401619 | 14/15 (93%) |
| 15:78401661 | 10/14 (71%) |
| 15:78401929 | 7/10 (70%) |
| 15:78402030 | 17/17 (100%) |
| 15:78402178 | 8/8 (100%) |
| 15:78402262 | 8/8 (100%) |
| 15:78402438 | 13/17 (76%) |
| 15:78402482 | 13/17 (76%) |
| 15:78402530 | 18/19 (94%) |
| 15:78402560 | 14/15 (93%) |
| 15:78402660 | 12/15 (80%) |
| 15:78402689 | 14/16 (87%) |
| 15:78402760 | 12/13 (92%) |
| 15:78402978 | 8/12 (66%) |
| 15:78403248 | 7/12 (58%) |
| 15:78403439 | 10/14 (71%) |
| 15:78403453 | 8/13 (61%) |
| 15:78403580 | 13/18 (72%) |
| 15:78403828 | 9/10 (90%) |
| 15:78403838 | 8/11 (72%) |
| 15:78403857 | 11/14 (78%) |
| Chr: bp | Methylated reads |
| 15:78404066 | 16/21 (76%) |
| 15:78404444 | 6/7 (85%) |
| 15:78404637 | 10/14 (71%) |
| 15:78404745 | 16/18 (88%) |
| 15:78404785 | 16/19 (84%) |
| 15:78404966 | 10/13 (76%) |
| 15:78404989 | 11/12 (91%) |
| 15:78405060 | 9/12 (75%) |
| 15:78405092 | 14/17 (82%) |
| 15:78405117 | 14/18 (77%) |
| 15:78405249 | 13/17 (76%) |
| 15:78405255 | 13/16 (81%) |
| 15:78405274 | 12/20 (60%) |
| 15:78405286 | 15/17 (88%) |
| 15:78405380 | 6/20 (30%) |
| 15:78405396 | 3/19 (15%) |
| 15:78405414 | 5/19 (26%) |
| 15:78405439 | 10/19 (52%) |
| 15:78405469 | 12/15 (80%) |
| 15:78405484 | 9/13 (69%) |
| 15:78405536 | 4/13 (30%) |
| 15:78405557 | 5/14 (35%) |
| 15:78405627 | 4/19 (21%) |
| 15:78405633 | 1/19 (5%) |
| 15:78405640 | 1/21 (4%) |
| 15:78405666 | 1/18 (5%) |
| 15:78405677 | 2/18 (11%) |
| 15:78405682 | 1/18 (5%) |
| 15:78405700 | 0/17 (0%) |
| 15:78405709 | 1/17 (5%) |
| 15:78405716 | 1/20 (5%) |
| 15:78405732 | 0/19 (0%) |
| 15:78405745 | 0/19 (0%) |
| 15:78405763 | 1/24 (4%) |
| 15:78405772 | 0/31 (0%) |
| 15:78405783 | 1/28 (3% |
| 15:78405807 | 0/23 (0%) |
| 15:78405847 | 0/24 (0%) |
| 15:78405859 | 0/22 (0%) |
| 15:78405904 | 0/15 (0%) |
| 15:78405911 | 1/14 (7%) |
| 15:78405925 | 0/10 (0%) |
| 15:78405956 | 0/10 (0%) |
| 15:78405964 | 0/10 (0%) |
| 15:78406048 | 1/14 (7%) |
| 15:78406051 | 1/15 (6%) |
| 15:78406087 | 0/18 (0%) |
| 15:78406102 | 1/22 (4%) |
| 15:78406202 | 0/23 (0%) |
| 15:78406210 | 0/24 (0%) |
| 15:78406237 | 0/25 (0%) |
| 15:78406282 | 1/19 (5%) |
| 15:78406316 | 0/15 (0%) |
| 15:78406333 | 0/12 (0%) |
| 15:78406348 | 0/8 (0%) |
| 15:78406353 | 0/8 (0%) |
| 15:78406366 | 0/8 (0%) |
| 15:78406390 | 0/15 (0%) |
| 15:78406398 | 0/14 (0%) |
