# Supplementary material for: Construction of an integrative regulatory element and variation map of the murine Tst locus
Source: BMC Genet. 2016 Jun 11;17:77. doi: 10.1186/s12863-016-0381-6 (PMC4902921; doi:10.1186/s12863-016-0381-6)
Supplement: Additional file 7: Table S7. — Sites enriched for marks of transcription factor binding sites. (DOCX 23 kb) [file 12863_2016_381_MOESM7_ESM.docx]

Table S7. Sites enriched for marks of transcription factor binding sites.

|  | Chr:bp | TF | Cell type |
| --- | --- | --- | --- |
| Ensembl | 15:78403183-78403832 | *Esrrb* | ES |
|  | 15:78405663-78406021 | *Zfx* | ES |
|  | 15:78405689-78406582 | *E2F1* | ES |
|  | 15:78405805-78406285 | *Klf4* | ES |
|  | 15:78406153-78406540 | *NELFe* | MEL |
| Alibaba 2.1 | 15:78405026-78405035 | *RAP1* |  |
|  | 15:78405034-78405043 | *MCM1* |  |
|  | 15:78405034-78405043 | *SRF* |  |
|  | 15:78405045-78405054 | *GR* |  |
|  | 15:78405056-78405065 | *YY1* |  |
|  | 15:78405065-78405074 | *NF-1* |  |
|  | 15:78405073-78405083 | *Sp1* |  |
|  | 15:78405114-78405128 | *Sp1* |  |
|  | 15:78405162-78405171 | *Sp1* |  |
|  | 15:78405186-78405195 | *AP-2alph* |  |
|  | 15:78405188-78405197 | *Sp1* |  |
|  | 15:78405201-78405212 | *C/EBPbeta* |  |
|  | 15:78405248-78405257 | *Sp1* |  |
|  | 15:78405257-78405266 | *C/EBPalp* |  |
|  | 15:78405259-78405268 | *NF-1* |  |
|  | 15:78405277-78405289 | *Sp1* |  |
|  | 15:78405293-78405302 | *HEB* |  |
|  | 15:78405293-78405302 | *MyoD* |  |
|  | 15:78405300-78405309 | *HSF* |  |
|  | 15:78405307-78405316 | *TBP* |  |
|  | 15:78405317-78405326 | *c-Rel* |  |
|  | 15:78405324-78405333 | *HSTF* |  |
|  | 15:78405326-78405335 | *T3R* |  |
|  | 15:78405326-78405335 | *T3R-beta* |  |
|  | 15:78405330-78405339 | *Sp1* |  |
|  | 15:78405343-78405352 | *C/EBPalp* |  |
|  | 15:78405350-78405359 | *Sp1* |  |
|  | 15:78405354-78405363 | *GATA-1* |  |
|  | 15:78405387-78405396 | *C/EBPalp* |  |
|  | 15:78405409-78405418 | *USF* |  |
|  | 15:78405462-78405471 | *ADR1* |  |
|  | 15:78405468-78405477 | *Sp1* |  |
|  | 15:78405472-78405481 | *NF-1* |  |
|  | 15:78405486-78405495 | *SGF-1* |  |
|  | 15:78405501-78405510 | *NF-1* |  |
|  | 15:78405513-78405525 | *Sp1* |  |
|  | 15:78405515-78405524 | *AP-2* |  |
|  | 15:78405516-78405525 | *AP-2* |  |
|  | 15:78405519-78405528 | *ER* |  |
|  | 15:78405520-78405529 | *ATF-a* |  |
|  | 15:78405524-78405533 | *Ttx* |  |
|  | 15:78405545-78405554 | *USF* |  |
|  | 15:78405602-78405611 | *Sp1* |  |
|  | 15:78405602-78405611 | *NF-1* |  |
|  | 15:78405616-78405625 | *Oct-1* |  |
|  | 15:78405666-78405675 | *AP-2alph* |  |
|  | 15:78405673-78405682 | *EmBP-1* |  |
|  | 15:78405675-78406584 | *USF* |  |
|  | 15:78405682-78408691 | *Sp1* |  |
|  | 15:78405707-78405717 | *Sp1* |  |
|  | 15:78405709-78405718 | *AP-2alph* |  |
|  | 15:78405749-78405760 | *Sp1* |  |
|  | 15:78405754-78405763 | *AP-2* |  |
|  | 15:78405755-78405764 | *YY1* |  |

|  | Chr:bp | TF |
| --- | --- | --- |
| Alibaba 2.1 | 15:78405762-78405771 | *NF-1* |
|  | 15:78405802-78405813 | *Sp1* |
|  | 15:78405848-78405857 | *c-Jun* |
|  | 15:78405857-78405866 | *Sp1* |
|  | 15:78405892-78405903 | *Sp1* |
|  | 15:78405907-78405917 | *Sp1* |
|  | 15:78405914-78405923 | *Sp1* |
|  | 15:78405920-78405929 | *Egr-1* |
|  | 15:78405920-78405931 | *Sp1* |
|  | 15:78405941-78405950 | *YY1* |
|  | 15:78405944-78405953 | *PTF1-beta* |
|  | 15:78405983-78405992 | *AP-1* |
|  | 15:78405992-78406001 | *Sp1* |
|  | 15:78406002-78406011 | *NF-1* |
|  | 15:78406004-78406013 | *TEC1* |
|  | 15:78406006-78406015 | *HNF-1C* |
|  | 15:78406010-78406019 | *Olf-1* |
|  | 15:78406016-78406025 | *NF-kappa* |
|  | 15:78406027-78406039 | *Sp1* |
|  | 15:78406028-78406037 | *CACCC-bi* |
|  | 15:78406030-78406039 | *CPE-bind* |
|  | 15:78406033-78406047 | *Sp1* |
|  | 15:78406045-78406054 | *Krox-20* |
|  | 15:78406046-78406055 | *AP-2* |
|  | 15:78406046-78406059 | *Sp1* |
|  | 15:78406054-78406063 | *ICSBP* |
|  | 15:78406069-78406082 | *Sp1* |
|  | 15:78406075-78406084 | *MyoD* |
|  | 15:78406075-78406084 | *RXR-beta* |
|  | 15:78406076-78406085 | *E1* |
|  | 15:78406076-78406085 | *Max1* |
|  | 15:78406079-78406092 | *Sp1* |
|  | 15:78406128-78406142 | *Sp1* |
|  | 15:78406136-78406145 | *c-Jun* |
|  | 15:78406161-78406170 | *TEC1* |
|  | 15:78406164-78406173 | *NF-kappa* |
|  | 15:78406169-78406178 | *HNF-3* |
|  | 15:78406180-78406189 | *c-Jun* |
|  | 15:78406211-78406220 | *Sp1* |
|  | 15:78406234-78406243 | *ICSBP* |
|  | 15:78406245-78406254 | *NF-1* |
|  | 15:78406262-78406272 | *HSE-bind* |
|  | 15:78406263-78406270 | *Pit-1a* |
|  | 15:78406271-78406282 | *C/EBPalpha* |
|  | 15:78406275-78406284 | *HOXA4* |
|  | 15:78406300-78406309 | *Sp1* |
|  | 15:78406302-78406311 | *RXR-alpha* |
|  | 15:78406334-78406343 | *Sp1* |
|  | 15:78406341-78406353 | *Sp1* |
|  | 15:78406344-78406353 | *represso* |
|  | 15:78406377-78406386 | *Sp1* |
|  | 15:78406379-78406389 | *NF-1* |
| MotifMap | 15:78406322-78406329 | *MFZ1* |
|  | 15:78406349-78406355 | *Arnt* |
|  | 15:78406348-78406355 | *MyC* |
|  | 15:78406348-78406356 | *MyC* |
|  | 15:78406348-78406356 | *CLOCK-BMA* |
|  | 15:78406368-78406374 | *HNF4* |
|  | 15:78406377-78406383 | *Arnt* |
