# Supplementary material for: Construction of an integrative regulatory element and variation map of the murine Tst locus
Source: BMC Genet. 2016 Jun 11;17:77. doi: 10.1186/s12863-016-0381-6 (PMC4902921; doi:10.1186/s12863-016-0381-6)
Supplement: Additional file 8: Table S8. — Predicted miRNA target sites. (DOCX 22 kb) [file 12863_2016_381_MOESM8_ESM.docx]

Table S8. Predicted miRNA target sites.

|  | Chr:bp | miRNA |
| --- | --- | --- |
| Microcosm | 15:78399625-78399645 | mmu-miR-10a |
|  | 15:78399625-78399645 | mmu-miR-10b |
|  | 15:78399632-78399651 | mmu-miR-338-5p |
|  | 15:78399627-78399647 | mmu-miR-582-3p |
|  | 15:78399649-78399668 | mmu-miR-18a |
|  | 15:78399657-78399676 | mmu-miR-139-5p |
|  | 15:78399660-78399677 | mmu-miR-761 |
|  | 15:78399660-78399677 | mmu-miR-214 |
|  | 15:78399662-78399686 | hsa-miR-657 |
|  | 15:78399664-78399683 | mmu-miR-154 |
|  | 15:78399667-78399683 | mmu-miR-485 |
|  | 15:78399679-78399693 | mmu-miR-351 |
|  | 15:78399679-78399696 | mmu-miR-670 |
|  | 15:78399679-78399691 | mmu-miR-691 |
|  | 15:78399691-78399713 | hsa-miR-892b |
|  | 15:78399698-78399705 | mmu-miR-695 |
|  | 15:78399708-78399731 | mmu-miR-298 |
|  | 15:78399709-78399726 | mmu-miR-877 |
|  | 15:78399719-78399728 | mmu-miR-604 |
|  | 15:78399720-78399731 | mmu-miR-647 |
| miRDB | 15:78399626-78399632 | mmu-miR-339-5p |
|  | 15:78399625-78399631 | mmu-miR-10a-5p |
|  | 15:78399709-78399715 | mmu-miR-298-5p |
|  | 15:78399707-78399713 | mmu-miR-5107-5p |
|  | 15:78399693-78399699 | mmu-miR-664-5p |
| miRecords | 15:78399589-78399607 | mmu-miR-705 |
|  | 15:78399625-78399642 | mmu-miR-10a |
|  | 15:78399625-78399642 | mmu-miR-10b |
|  | 15:78399626-78399647 | mmu-miR-339-5p |
|  | 15:78399632-78399651 | mmu-miR-338-5p |
|  | 15:78399649-78399668 | mmu-miR-18a |
|  | 15:78399660-78399678 | mmu-miR-214 |
|  | 15:78399660-78399677 | mmu-miR-761 |
|  | 15:78399679-78399700 | mmu-miR-691 |
|  | 15:78399679-78399700 | mmu-miR-670 |
|  | 15:78399709-78399726 | mmu-miR-877 |
| miRWalk | 15:78399565-78399571 | mmu-miR-124-3p |
|  | 15:78399583-78399589 | mmu-miR-877-5p |
|  | 15:78399601-78399607 | mmu-miR-709 |
|  | 15:78399609-78399618 | mmu-miR-335-5p |
|  | 15:78399625-78399631 | mmu-miR-10a-5p |
|  | 15:78399625-78399631 | mmu-miR-10b-5p |
|  | 15:78399626-78399632 | mmu-miR-339-5p |

|  | Chr:bp | miRNA |
| --- | --- | --- |
| miRWalk | 15:78399660-78399667 | mmu-miR-214-3p |
|  | 15:78399660-78399667 | mmu-miR-761 |
|  | 15:78399665-78399671 | mmu-miR-1193-3p |
|  | 15:78399679-78399685 | mmu-miR-351-5p |
|  | 15:78399679-78399685 | mmu-miR-125a-5p |
|  | 15:78399679-78399685 | mmu-miR-125b-5p |
|  | 15:78399679-78399685 | mmu-miR-670-5p |
|  | 15:78399690-78399696 | mmu-miR-193b-3p |
|  | 15:78399690-78399696 | mmu-miR-193a-3p |
|  | 15:78399699-78399705 | mmu-miR-695 |
|  | 15:78399708-78399718 | mmu-miR-298-5p |
|  | 15:78399716-78399722 | mmu-miR-764-3p |
|  | 15:78399718-78399724 | mmu-miR-485-5p |
|  | 15:78399720-78399726 | mmu-miR-666-3p |
|  | 15:78399721-78399727 | mmu-miR-191-3p |
|  | 15:78399737-78399744 | mmu-miR-665-3p |
|  | 15:78399750-78399757 | mmu-miR-331-3p |
|  | 15:78399767-78399774 | mmu-miR-330-5p |
|  | 15:78399815-78399824 | mmu-miR-693-5p |
|  | 15:78399844-78399851 | mmu-miR-326-3p |
|  | 15:78399844-78399851 | mmu-miR-330-5p |
|  | 15:78399863-78399870 | mmu-miR-34c-5p |
|  | 15:78399863-78399870 | mmu-miR-449c-5p |
|  | 15:78399863-78399870 | mmu-miR-34b-5p |
|  | 15:78399863-78399870 | mmu-miR-449b |
|  | 15:78399893-78399907 | mmu-miR-423-5p |
|  | 15:78399930-78399937 | mmu-miR-183-5p |
|  | 15:78405257-78405264 | mmu-miR-669a-5p |
|  | 15:78405296-78405303 | mmu-miR-763 |
|  | 15:78405385-78408392 | mmu-miR-760-3p |
|  | 15:78405418-78405425 | mmu-miR-328-3p |
|  | 15:78405423-78405430 | mmu-miR-712-5p |
|  | 15:78405514-78405521 | mmu-miR-22-3p |
|  | 15:78405549-78405556 | mmu-miR-466a-5p |
|  | 15:78405549-78405556 | mmu-miR-1187 |
|  | 15:78405549-78405556 | mmu-miR-466b-5p |
|  | 15:78405549-78405556 | mmu-miR-466c-5p |
|  | 15:78405571-78405578 | mmu-miR-453 |
|  | 15:78405594-78405601 | mmu-miR-770-3p |
|  | 15:78405836-78405843 | mmu-miR-223-3p |
|  | 15:78405841-78405847 | mmu-miR-1904 |
|  | 15:78405846-78405856 | mmu-miR-712-3p |
|  | 15:78405851-78405858 | mmu-miR-412-3p |
