# Supplementary material for: Construction of an integrative regulatory element and variation map of the murine Tst locus
Source: BMC Genet. 2016 Jun 11;17:77. doi: 10.1186/s12863-016-0381-6 (PMC4902921; doi:10.1186/s12863-016-0381-6)
Supplement: Additional file 9: Table S9. — Merged mouse single nucleotide polymorphisms (SNP) from Ensembl and Mouse genomes project (Wellcome Trust Sanger Institute). (DOCX 63 kb) [file 12863_2016_381_MOESM9_ESM.docx]

Table S9. Merged mouse single nucleotide polymorphisms (SNP) from Ensembl and Mouse genomes project (Wellcome Trust Sanger Institute).

| Gene | dbSNP | Chr: bp | Alleles | Type | Resulting Amino Acid | AA coordinate | Variation effect on protein function | Reference | 129P2/OlaHsd | AKR/J | BALB/cJ | C3H/HeJ | C57BL/6NJ | CAST/EiJ | CBA/J | DBA/2J | FVB/NJ | LP/J | NOD/ShiLtJ | NZO/HlLtJ | PWK/PhJ | SPRET/EiJ | WSB/EiJ |
| --- | --- | --- | --- | --- | --- | --- | --- | --- | --- | --- | --- | --- | --- | --- | --- | --- | --- | --- | --- | --- | --- | --- | --- |
| *1700061J05Rik* | rs251522179 | 15:78399022 | T/C | Downstream gene variant | - | - | - | T | - | - | - | - | - | - | - | - | - | - | - | - | - | C | - |
| *1700061J05Rik* | rs220621086 | 15:78399052 | G/A | Downstream gene variant | - | - | - | G | - | - | - | - | - | - | - | - | - | - | - | - | - | A | - |
| *1700061J05Rik* | rs240914706 | 15:78399063 | T/C | Downstream gene variant | - | - | - | T | - | - | - | - | - | - | - | - | - | - | - | - | - | C | - |
| *1700061J05Rik* | rs6373036 | 15:78399065 | A/G | Downstream gene variant | - | - | - | A | - | - | - | - | - | - | - | - | - | - | - | - | G | G | - |
| *1700061J05Rik* | rs226314457 | 15:78399102 | A/T | Downstream gene variant | - | - | - | A | - | - | - | - | - | - | - | - | - | - | - | - | - | T | - |
| *1700061J05Rik* | rs248474782 | 15:78399105 | C/G | Downstream gene variant | - | - | - | C | - | - | - | - | - | - | - | - | - | - | - | - | - | G | - |
| *1700061J05Rik* | rs264697801 | 15:78399117 | C/T | Downstream gene variant | - | - | - | C | - | - | - | - | - | - | - | - | - | - | - | - | - | T | - |
| *1700061J05Rik* | rs221285559 | 15:78399118 | A/G | Downstream gene variant | - | - | - | A | - | - | - | - | - | - | - | - | - | - | - | - | G | G | - |
| *1700061J05Rik* | rs237398739 | 15:78399129 | G/A | Downstream gene variant | - | - | - | G | - | - | - | - | - | - | - | - | - | - | - | - | - | A | - |
| *1700061J05Rik* | rs256203372 | 15:78399135 | G/T | Downstream gene variant | - | - | - | G | - | - | - | - | - | - | - | - | - | - | - | - | - | T | - |
| *1700061J05Rik* | rs226824666 | 15:78399145 | A/G | Downstream gene variant | - | - | - | A | - | - | - | - | - | - | - | - | - | - | - | - | G | G | - |
| *1700061J05Rik* | rs245996149 | 15:78399157 | A/G | Downstream gene variant | - | - | - | A | - | - | - | - | - | - | - | - | - | - | - | - | G | G | - |
| *1700061J05Rik* | rs263852494 | 15:78399178 | A/G | Downstream gene variant | - | - | - | A | - | - | - | - | - | - | - | - | - | - | - | - | G | - | - |
| *1700061J05Rik* | rs234599937 | 15:78399302 | A/G | Downstream gene variant | - | - | - | A | - | - | - | - | - | - | - | - | - | - | - | - | - | G | - |
| *1700061J05Rik* | rs248929150 | 15:78399303 | G/A | Downstream gene variant | - | - | - | G | - | - | - | - | - | - | - | - | - | - | - | - | - | A | - |
| *1700061J05Rik* | rs213004608 | 15:78399310 | C/G | Downstream gene variant | - | - | - | C | - | - | - | - | - | - | - | - | - | - | - | - | - | G | - |
| *1700061J05Rik* | rs31534685 | 15:78399324 | G/A | Downstream gene variant | - | - | - | G | - | - | - | - | - | - | - | - | - | - | - | - | A | A | - |
| *1700061J05Rik* | rs245239061 | 15:78399347 | A/T | Downstream gene variant | - | - | - | A | - | - | - | - | - | - | - | - | - | - | - | - | T | - | - |
| *1700061J05Rik* | rs214707841 | 15:78399348 | T/A | Downstream gene variant | - | - | - | T | - | - | - | - | - | - | - | - | - | - | - | - | A | - | - |
| *1700061J05Rik* | rs234352724 | 15:78399395 | T/C | Downstream gene variant | - | - | - | T | - | - | - | - | - | - | - | - | - | - | - | - | - | C | - |
| *1700061J05Rik* | rs263571566 | 15:78399546 | G/A | Downstream gene variant | - | - | - | G | - | - | - | - | - | - | - | - | - | - | - | - | - | A | - |
| *Tst* | rs225783190 | 15:78399558 | C/T | 3 prime UTR variant | - | - | - | C | - | - | - | - | - | - | - | - | - | - | - | - | - | T | - |
| *Tst* | rs31534689 | 15:78399636 | A/C | 3 prime UTR variant | - | - | - | A | - | - | - | - | - | C | - | - | - | - | - | - | C | C | C |
| *Tst* | rs255601122 | 15:78399888 | G/T | Synonymous variant | A | 246 | - | G | - | - | - | - | - | - | - | - | - | - | - | - | - | T | - |
| *Tst* | rs221276231 | 15:78399971 | T/C | Missense variant | K/E | 219 | 1 | T | - | - | - | - | - | - | - | - | - | - | - | - | - | C | - |
| *Tst* | rs237807264 | 15:78400034 | G/C | Splice region variant | - | - | - | G | - | - | - | - | - | - | - | - | - | - | - | - | - | C | - |
| *Tst* | rs256157951 | 15:78400043 | A/C | Intron variant | - | - | - | A | - | - | - | - | - | - | - | - | - | - | - | - | - | C | - |
| *Tst* | rs220652493 | 15:78400111 | G/A | Intron variant | - | - | - | G | - | - | - | - | - | - | - | - | - | - | - | - | - | A | - |
| *Tst* | rs239954548 | 15:78400161 | C/T | Intron variant | - | - | - | C | - | - | - | - | - | - | - | - | - | - | - | - | - | T | - |
| *Tst* | rs265923749 | 15:78400253 | G/A | Intron variant | - | - | - | G | - | - | - | - | - | - | - | - | - | - | - | - | - | A | - |
| *Tst* | rs233527510 | 15:78400318 | T/C | Intron variant | - | - | - | T | - | - | - | - | - | - | - | - | - | - | - | - | - | C | - |
| *Tst* | rs251994838 | 15:78400343 | G/T | Intron variant | - | - | - | G | - | - | - | - | - | - | - | - | - | - | - | - | - | - | T |
| *Tst* | rs261791516 | 15:78400398 | C/T | Intron variant | - | - | - | C | - | - | - | - | - | - | - | - | - | - | - | - | - | T | - |
| *Tst* | rs225533569 | 15:78400402 | G/A | Intron variant | - | - | - | G | - | - | - | - | - | - | - | - | - | - | - | - | - | A | - |
| *Tst* | rs245557674 | 15:78400421 | A/G | Intron variant | - | - | - | A | - | - | - | - | - | - | - | - | - | - | - | - | - | G | - |
| *Tst* | rs31534691 | 15:78400429 | T/C | Intron variant | - | - | - | T | - | - | - | - | - | C | - | - | - | - | - | - | C | C | C |
| *Tst* | rs234258563 | 15:78400430 | G/T | Intron variant | - | - | - | G | - | - | - | - | - | - | - | - | - | - | - | - | - | T | - |
| *Tst* | rs253495918 | 15:78400492 | C/A | Intron variant | - | - | - | C | - | - | - | - | - | - | - | - | - | - | - | - | A | - | - |

*Table 9 (continued)*

| Gene | dbSNP | Chr: bp | Alleles | Type | Resulting Amino Acid | AA coordinate | Variation effect on protein function | Reference | 129P2/OlaHsd | AKR/J | BALB/cJ | C3H/HeJ | C57BL/6NJ | CAST/EiJ | CBA/J | DBA/2J | FVB/NJ | LP/J | NOD/ShiLtJ | NZO/HlLtJ | PWK/PhJ | SPRET/EiJ | WSB/EiJ |
| --- | --- | --- | --- | --- | --- | --- | --- | --- | --- | --- | --- | --- | --- | --- | --- | --- | --- | --- | --- | --- | --- | --- | --- |
| *Tst* | rs218556451 | 15:78400501 | C/T | Intron variant | - | - | - | C | - | - | - | - | - | - | - | - | - | - | - | - | T | - | - |
| *Tst* | rs240738657 | 15:78400649 | T/C | Intron variant | - | - | - | T | - | - | - | - | - | - | - | - | - | - | - | - | - | C | - |
| *Tst* | rs213008426 | 15:78400915 | A/G | Intron variant | - | - | - | A | - | - | - | - | - | G | - | - | - | - | - | - | - | - | - |
| *Tst* | rs231535460 | 15:78400939 | T/A | Intron variant | - | - | - | T | - | - | - | - | - | - | - | - | - | - | - | - | - | A | - |
| *Tst* | rs250651863 | 15:78401108 | A/T | Intron variant | - | - | - | A | - | - | - | - | - | - | - | - | - | - | - | - | - | T | - |
| *Tst* | rs220616367 | 15:78401110 | G/A | Intron variant | - | - | - | G | - | - | - | - | - | - | - | - | - | - | - | - | - | A | - |
| *Tst* | rs248806009 | 15:78401117 | G/A | Intron variant | - | - | - | G | - | - | - | - | - | - | - | - | - | - | - | - | - | A | - |
| *Tst* | rs31535585 | 15:78401140 | G/A | Intron variant | - | - | - | G | - | - | - | - | - | A | - | - | - | - | - | - | - | - | - |
| *Tst* | rs227093259 | 15:78401160 | C/T | Intron variant | - | - | - | C | - | - | - | - | - | - | - | - | - | - | - | - | - | - | T |
| *Tst* | rs241211319 | 15:78401228 | G/T | Intron variant | - | - | - | G | - | - | - | - | - | T | - | - | - | - | - | - | T | - | - |
| *Tst* | rs264774126 | 15:78401239 | A/G | Intron variant | - | - | - | A | - | - | - | - | - | G | - | - | - | - | - | - | G | G | - |
| *Tst* | rs225441409 | 15:78401240 | C/A | Intron variant | - | - | - | C | - | - | - | - | - | A | - | - | - | - | - | - | A | A | - |
| *Tst* | rs239435067 | 15:78401306 | G/A | Intron variant | - | - | - | G | - | - | - | - | - | - | - | - | - | - | - | - | - | A | - |
| *Tst* | rs258900594 | 15:78401360 | T/C | Intron variant | - | - | - | T | - | - | - | - | - | C | - | - | - | - | - | - | - | C | - |
| *Tst* | rs31535587 | 15:78401371 | G/A | Intron variant | - | - | - | G | - | - | - | - | - | A | - | - | - | - | - | - | - | - | - |
| *Tst* | rs258576442 | 15:78401373 | G/A | Intron variant | - | - | - | G | - | - | - | - | - | - | - | - | - | - | - | - | - | A | - |
| *Tst* | rs31535589 | 15:78401383 | C/T | Intron variant | - | - | - | C | - | - | - | - | - | - | - | - | - | - | - | - | T | - | - |
| *Tst* | rs235722146 | 15:78401384 | G/A | Intron variant | - | - | - | G | - | - | - | - | - | - | - | - | - | - | - | - | - | A | - |
| *Tst* | rs249478062 | 15:78401704 | G/A | Intron variant | - | - | - | G | - | - | - | - | - | - | - | - | - | - | - | - | A | - | - |
| *Tst* | rs212918899 | 15:78401735 | A/G | Intron variant | - | - | - | A | - | - | - | - | - | G | - | - | - | - | - | - | - | - | - |
| *Tst* | rs231503123 | 15:78401736 | C/T | Intron variant | - | - | - | C | - | - | - | - | - | - | - | - | - | - | - | - | - | T | - |
| *Tst* | rs31535591 | 15:78401737 | A/G | Intron variant | - | - | - | A | - | - | - | - | - | G | - | - | - | - | - | - | G | G | - |
| *Tst* | rs215344152 | 15:78401749 | T/C | Intron variant | - | - | - | T | - | - | - | - | - | C | - | - | - | - | - | - | - | - | - |
| *Tst* | rs238867697 | 15:78401752 | C/T | Intron variant | - | - | - | C | - | - | - | - | - | T | - | - | - | - | - | - | - | - | - |
| *Tst* | rs263688106 | 15:78402013 | T/G | Intron variant | - | - | - | T | - | - | - | - | - | - | - | - | - | - | - | - | - | G | - |
| *Tst* | rs226606707 | 15:78402048 | C/T | Intron variant | - | - | - | C | - | - | - | - | - | - | - | - | - | - | - | - | - | T | - |
| *Tst* | rs243923307 | 15:78402242 | A/G | Intron variant | - | - | - | A | - | - | - | - | - | - | - | - | - | - | - | - | - | G | - |
| *Tst* | rs250413862 | 15:78402250 | T/C | Intron variant | - | - | - | T | - | - | - | - | - | C | - | - | - | - | - | - | C | C | - |
| *Tst* | rs219260437 | 15:78402272 | A/G | Intron variant | - | - | - | A | - | - | - | - | - | - | - | - | - | - | - | - | - | G | - |
| *Tst* | rs239395161 | 15:78402290 | C/T | Intron variant | - | - | - | C | - | - | - | - | - | - | - | - | - | - | - | - | - | T | - |
| *Tst* | rs31535593 | 15:78402322 | G/A | Intron variant | - | - | - | G | - | - | - | - | - | - | - | - | - | - | - | - | A | - | - |
| *Tst* | rs227909723 | 15:78402420 | G/A | Intron variant | - | - | - | G | - | - | - | - | - | A | - | - | - | - | - | - | A | - | - |
| *Tst* | rs247207285 | 15:78402454 | G/A | Intron variant | - | - | - | G | - | - | - | - | - | - | - | - | - | - | - | - | - | A | - |
| *Tst* | rs265987291 | 15:78402482 | C/T | Intron variant | - | - | - | C | - | - | - | - | - | - | - | - | - | - | - | - | - | T | - |
| *Tst* | rs234136097 | 15:78402597 | C/T | Intron variant | - | - | - | C | - | - | - | - | - | - | - | - | - | - | - | - | - | T | - |
| *Tst* | rs31536425 | 15:78402613 | A/G | Intron variant | - | - | - | A | - | - | - | - | - | G | - | - | - | - | - | - | G | - | - |
| *Tst* | rs213275186 | 15:78402660 | C/T | Intron variant | - | - | - | C | - | - | - | - | - | - | - | - | - | - | - | - | - | T | - |
| *Tst* | rs225739160 | 15:78402686 | C/G | Intron variant | - | - | - | C | - | - | - | - | - | - | - | - | - | - | - | - | - | G | - |

*Table 9 (continued)*

| Gene | dbSNP | Chr: bp | Alleles | Type | Resulting Amino Acid | AA coordinate | Variation effect on protein function | Reference | 129P2/OlaHsd | AKR/J | BALB/cJ | C3H/HeJ | C57BL/6NJ | CAST/EiJ | CBA/J | DBA/2J | FVB/NJ | LP/J | NOD/ShiLtJ | NZO/HlLtJ | PWK/PhJ | SPRET/EiJ | WSB/EiJ |
| --- | --- | --- | --- | --- | --- | --- | --- | --- | --- | --- | --- | --- | --- | --- | --- | --- | --- | --- | --- | --- | --- | --- | --- |
| *Tst* | rs244606961 | 15:78402690 | G/A | Intron variant | - | - | - | G | - | - | - | - | - | - | - | - | - | - | - | - | - | A | - |
| *Tst* | rs31536427 | 15:78402754 | T/C | Intron variant | - | - | - | T | - | - | - | - | - | C | - | - | - | - | - | - | C | C | - |
| *Tst* | rs241298782 | 15:78402780 | T/C | Intron variant | - | - | - | T | - | - | - | - | - | - | - | - | - | - | - | - | - | C | - |
| *Tst* | rs254100397 | 15:78402789 | G/A | Intron variant | - | - | - | G | - | - | - | - | - | - | - | - | - | - | - | - | - | A | - |
| *Tst* | rs218879758 | 15:78402797 | A/G | Intron variant | - | - | - | A | - | - | - | - | - | - | - | - | - | - | - | - | - | G | - |
| *Tst* | rs234571147 | 15:78402810 | G/A | Intron variant | - | - | - | G | - | - | - | - | - | - | - | - | - | - | - | - | - | A | - |
| *Tst* | rs250370919 | 15:78402819 | G/C | Intron variant | - | - | - | G | - | - | - | - | - | - | - | - | - | - | - | - | - | C | - |
| *Tst* | rs31536429 | 15:78402839 | G/A | Intron variant | - | - | - | G | - | - | - | - | - | A | - | - | - | - | - | - | A | - | - |
| *Tst* | rs232865603 | 15:78403032 | T/A | Intron variant | - | - | - | T | - | - | - | - | - | - | - | - | - | - | - | - | - | A | - |
| *Tst* | rs252500175 | 15:78403063 | T/C | Intron variant | - | - | - | T | - | - | - | - | - | - | - | - | - | - | - | - | - | C | - |
| *Tst* | rs224674506 | 15:78403101 | G/A | Intron variant | - | - | - | G | - | - | - | - | - | - | - | - | - | - | - | - | A | - | - |
| *Tst* | rs31536431 | 15:78403104 | C/T | Intron variant | - | - | - | C | - | - | - | - | - | T | - | - | - | - | - | - | T | - | - |
| *Tst* | rs252857874 | 15:78403209 | T/- | Intron variant | - | - | - | T | - | - | - | - | - | / | - | - | - | - | - | - | / | - | / |
| *Tst* | rs264325545 | 15:78403297 | T/C | Intron variant | - | - | - | T | - | - | - | - | - | C | - | - | - | - | - | - | C | C | - |
| *Tst* | rs227453977 | 15:78403326 | T/G | Intron variant | - | - | - | T | - | - | - | - | - | G | - | - | - | - | - | - | G | G | - |
| *Tst* | rs236135035 | 15:78403379 | C/T | Intron variant | - | - | - | C | - | - | - | - | - | - | - | - | - | - | - | - | - | T | - |
| *Tst* | rs254895938 | 15:78403382 | C/T | Intron variant | - | - | - | C | - | - | - | - | - | - | - | - | - | - | - | - | - | T | - |
| *Tst* | rs225701420 | 15:78403440 | G/C | Intron variant | - | - | - | G | - | - | - | - | - | - | - | - | - | - | - | - | C | - | - |
| *Tst* | rs244922891 | 15:78403470 | G/A | Intron variant | - | - | - | G | - | - | - | - | - | - | - | - | - | - | - | - | - | A | - |
| *Tst* | rs257269253 | 15:78403584 | C/T | Intron variant | - | - | - | C | - | - | - | - | - | T | - | - | - | - | - | - | - | - | - |
| *Tst* | rs232452949 | 15:78403602 | A/G | Intron variant | - | - | - | A | - | - | - | - | - | - | - | - | - | - | - | - | - | G | - |
| *Tst* | rs259334849 | 15:78403617 | A/G | Intron variant | - | - | - | A | - | - | - | - | - | - | - | - | - | - | - | - | - | G | - |
| *Tst* | rs219485441 | 15:78403811 | G/A | Intron variant | - | - | - | G | - | - | - | - | - | - | - | - | - | - | - | - | - | A | - |
| *Tst* | rs236512282 | 15:78403816 | C/T | Intron variant | - | - | - | C | - | - | - | - | - | - | - | - | - | - | - | - | - | T | - |
| *Tst* | rs244158757 | 15:78403820 | A/T | Intron variant | - | - | - | A | - | - | - | - | - | - | - | - | - | - | - | - | - | T | - |
| *Tst* | rs213570577 | 15:78403827 | C/T | Intron variant | - | - | - | C | - | - | - | - | - | - | - | - | - | - | - | - | - | T | - |
| *Tst* | rs232724534 | 15:78403965 | G/A | Intron variant | - | - | - | G | - | - | - | - | - | - | - | - | - | - | - | - | - | A | - |
| *Tst* | rs31536433 | 15:78403966 | G/A | Intron variant | - | - | - | G | - | - | - | - | - | - | - | - | - | - | - | - | A | - | - |
| *Tst* | rs224748275 | 15:78404010 | G/A | Intron variant | - | - | - | G | - | - | - | - | - | - | - | - | - | - | - | - | - | A | - |
| *Tst* | rs31537095 | 15:78404031 | A/G | Intron variant | - | - | - | A | - | - | - | - | - | G | - | - | - | - | - | - | - | - | - |
| *Tst* | rs31537096 | 15:78404144 | A/G | Intron variant | - | - | - | A | - | - | - | - | - | G | - | - | - | - | - | - | G | G | - |
| *Tst* | rs220077051 | 15:78404197 | C/T | Intron variant | - | - | - | C | - | - | - | - | - | - | - | - | - | - | - | - | - | T | - |
| *Tst* | rs236509514 | 15:78404205 | G/A | Intron variant | - | - | - | G | - | - | - | - | - | - | - | - | - | - | - | - | - | A | - |
| *Tst* | rs31532224 | 15:78404250 | A/C | Intron variant | - | - | - | A | - | - | - | - | - | C | - | - | - | - | - | - | - | - | - |
| *Tst* | rs219610985 | 15:78404286 | T/G | Intron variant | - | - | - | T | - | - | - | - | - | - | - | - | - | - | - | - | - | G | - |
| *Tst* | rs238714131 | 15:78404467 | T/C | Intron variant | - | - | - | T | - | - | - | - | - | C | - | - | - | - | - | - | - | - | - |
| *Tst* | rs263367155 | 15:78404582 | A/C | Intron variant | - | - | - | A | - | - | - | - | - | - | - | - | - | - | - | - | - | C | - |
| *Tst* | rs233149012 | 15:78404583 | G/A | Intron variant | - | - | - | G | - | - | - | - | - | - | - | - | - | - | - | - | - | A | - |

*Table 9 (continued)*

| Gene | dbSNP | Chr: bp | Alleles | Type | Resulting Amino Acid | AA coordinate | Variation effect on protein function | Reference | 129P2/OlaHsd | AKR/J | BALB/cJ | C3H/HeJ | C57BL/6NJ | CAST/EiJ | CBA/J | DBA/2J | FVB/NJ | LP/J | NOD/ShiLtJ | NZO/HlLtJ | PWK/PhJ | SPRET/EiJ | WSB/EiJ |
| --- | --- | --- | --- | --- | --- | --- | --- | --- | --- | --- | --- | --- | --- | --- | --- | --- | --- | --- | --- | --- | --- | --- | --- |
| *Tst* | rs253970453 | 15:78404725 | C/T | Intron variant | - | - | - | C | - | - | - | - | - | - | - | - | - | - | - | - | T | - | - |
| *Tst* | rs31532228 | 15:78404795 | T/G | Intron variant | - | - | - | T | - | - | - | - | - | - | - | - | - | - | - | - | G | G | - |
| *Tst* | rs224379604 | 15:78404823 | C/T | Intron variant | - | - | - | C | - | - | - | - | - | - | - | - | - | - | - | - | - | T | - |
| *Tst* | rs31532230 | 15:78404834 | G/C | Intron variant | - | - | - | G | - | - | - | - | - | C | - | - | - | - | - | - | - | - | - |
| *Tst* | rs213490846 | 15:78404960 | C/T | Intron variant | - | - | - | C | - | - | - | - | - | - | - | - | - | - | - | - | - | T | - |
| *Tst* | rs226460405 | 15:78404967 | G/A | Intron variant | - | - | - | G | - | - | - | - | - | - | - | - | - | - | - | - | - | A | - |
| *Tst* | rs246408586 | 15:78405024 | A/G | Intron variant | - | - | - | A | - | - | - | - | - | - | - | - | - | - | - | - | - | G | - |
| *Tst* | rs216632015 | 15:78405094 | C/A | Intron variant | - | - | - | C | - | - | - | - | - | - | - | - | - | - | - | - | - | A | - |
| *Tst* | rs31532232 | 15:78405103 | A/C | Intron variant | - | - | - | A | - | - | - | - | - | C | - | - | - | - | - | - | C | C | - |
| *Tst* | rs260631843 | 15:78405120 | A/C | Intron variant | - | - | - | A | - | - | - | - | - | - | - | - | - | - | - | - | - | C | - |
| *Tst* | rs211830862 | 15:78405342 | G/A | Synonymous variant | T | 164 | - | G | - | - | - | - | - | A | - | - | - | - | - | - | A | - | - |
| *Tst* | rs31532994 | 15:78405627 | C/T | Synonymous variant | S | 69 | - | C | - | - | - | - | - | T | - | - | - | - | - | - | - | - | - |
| *Mpst* | rs249476506 | 15:78405896 | G/A | Upstream gene variant | - | - | - | G | - | - | - | - | - | - | - | - | - | - | - | - | A | A | - |
| *Mpst* | rs219577774 | 15:78405936 | C/G | Upstream gene variant | - | - | - | C | - | - | - | - | - | - | - | - | - | - | - | - | - | G | - |
| *Mpst* | rs238674188 | 15:78406086 | G/A | Upstream gene variant | - | - | - | G | - | - | - | - | - | - | - | - | - | - | - | - | - | A | - |
| *Mpst* | rs263070511 | 15:78406124 | A/G | Upstream gene variant | - | - | - | A | - | - | - | - | - | - | - | - | - | - | - | - | - | G | - |
| *Mpst* | rs225309913 | 15:78406150 | T/A | Upstream gene variant | - | - | - | T | - | - | - | - | - | - | - | - | - | - | - | - | - | A | - |
| *Mpst* | rs250702506 | 15:78406197 | C/G | Upstream gene variant | - | - | - | C | - | - | - | - | - | - | - | - | - | - | - | - | G | - | - |
| *Mpst* | rs31532996 | 15:78406204 | C/T | Upstream gene variant | - | - | - | C | - | - | - | - | - | - | - | - | - | - | - | - | T | - | - |
| *Mpst* | rs224294200 | 15:78406322 | G/A | Upstream gene variant | - | - | - | G | - | - | - | - | - | - | - | - | - | - | - | - | - | A | - |
