# Supplementary material for: Construction of an integrative regulatory element and variation map of the murine Tst locus
Source: BMC Genet. 2016 Jun 11;17:77. doi: 10.1186/s12863-016-0381-6 (PMC4902921; doi:10.1186/s12863-016-0381-6)
Supplement: Additional file 10: Table S10. — Sequences of primers used in sequencing of Tst locus (region). (DOCX 17 kb) [file 12863_2016_381_MOESM10_ESM.docx]

Table S10: Sequences of primers used in sequencing of *Tst* locus (region).

|  | Primer name | Sequence (5' – 3') |
| --- | --- | --- |
| A | A1 | TCCTGTGCTCCCTCTACAGC |
|  | A2 | TCAGTGTGGAAACTGCTTGC |
|  | B1 | TGCCTAGCTTTGTGAGTCGT |
|  | B2 | GGTCCCCTTTGGTATGTGTG |
|  | C1 | GTGAGGTGGGCCAAAACTT |
|  | C2 | AAAACTGAAGCCCAAGACGA |
| B | A1.1 | TGGTTGAAGGTTGGCAAAGG |
|  | A1.2 | TGGCGGACAGAAACTTTGTG |
|  | A1.3 | TCCATAGTTAGAGGCCAGCC |
|  | A2.1 | ACTTTGGTGCTGTCTTGAGTC |
|  | A2.2 | CTTCTTTCTTCCCTGGGCAC |
|  | A2.3 | CATGTCAGTGCACCTCAGC |
|  | A2.4 | ACTGTAAGACCCAAGCCTGC |
|  | A2.5 | TGAAGGGAACTGGCTGGATT |
|  | A2.6 | CTTGGCTTGAGACATCCCAC |
|  | B1.1 | CTGTGTAACCCTGCCAGTGA |
|  | B1.2 | CTGATGTTCATGCCCCTGGA |
|  | B1.3 | CATTCAAGTCAGGAGGCACG |
|  | B1.4 | ACAAGGTCACAGGTACCCAG |
|  | B1.5 | TGGTGTGCATTTGTGTCCAG |
|  | B1.6 | CACTCTCCCTTCCTCCAGTT |
|  | B2.1 | CTCTGCCAACTCCATGTGTG |
|  | B2.2 | CCATGTACTGTGACAGGGCT |
|  | B2.3 | GGATCACAGAGAAGAGAGGCA |
|  | B2.4 | CCTCCTTCGTACATAGCAACC |
|  | C1.1 | TTCACAGACACTAGCCAGGG |
|  | C1.2 | CTTTGAAAACGGCTGGCTCT |
|  | C1.3 | CAGGAAAGAGACAGGCCTGA |
|  | C1.4 | CAGTCGCAAAAGTAGCTGGG |
|  | C2.1 | GAGCTGTGGTGAGGAGATCT |
|  | C2.2 | TGAAGCTCGAGGTGACACTG |
|  | C2.3 | CCTAGGGAATGTGCCAACCT |
|  | C2.4 | AGAAGCTGCCCAGGTCATC |
|  | C2.5 | AATAGGGGCAGCTTGTTAGC |
