# Supplementary figures and images for: Construction of an integrative regulatory element and variation map of the murine Tst locus
Source: BMC Genet. 2016 Jun 11;17:77. doi: 10.1186/s12863-016-0381-6 (PMC4902921; doi:10.1186/s12863-016-0381-6)

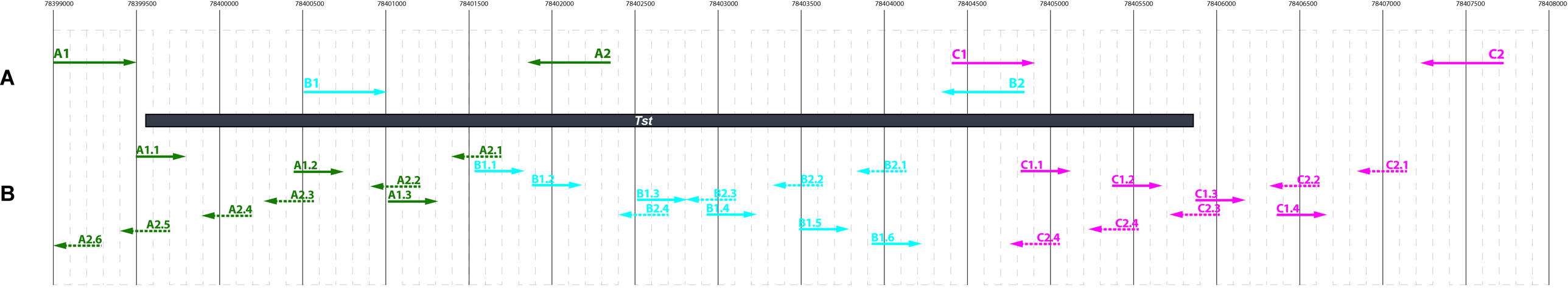

Supplement: Additional file 11: Figure S2. — Primers positions used in sequencing the Tst locus. A) Positions of primers used for amplification of ~ 3 kb segments (A, B, C) of Tst region. B) Location of PCR primers for detailed sequencing of A, B, and C segments. (JPG 4083 kb) [file 12863_2016_381_MOESM11_ESM.jpg]

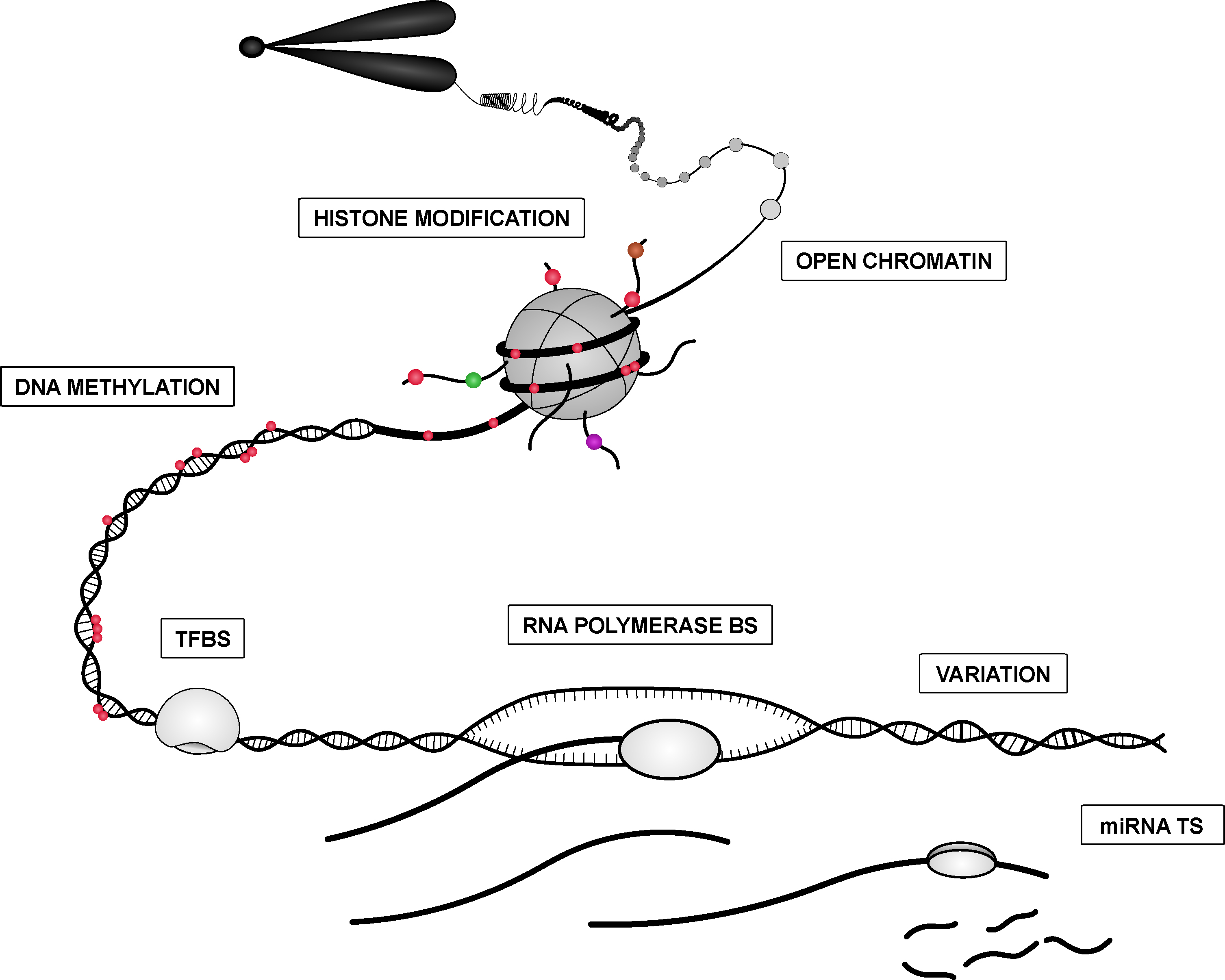

Supplement: Additional file 12: Figure S1. — Integrated gene transcription regulatory elements for atlas development. Schematic of elements affecting gene transcription and expression at the level of chromatin state (histone modifications, DNA methylation, chromatin accessibility), through transcription factors and RNA polymerase binding, variation impact and microRNAs miRNAs influence. (JPG 1035 kb) [file 12863_2016_381_MOESM12_ESM.jpg]
